# Uncovering disease mechanisms through network biology in the era of Next Generation Sequencing

**DOI:** 10.1038/srep24570

**Published:** 2016-04-15

**Authors:** Janet Piñero, Ariel Berenstein, Abel Gonzalez-Perez, Ariel Chernomoretz, Laura I. Furlong

**Affiliations:** 1 Research Programme on Biomedical Informatics (GRIB), Hospital del Mar Medical Research Institute (IMIM), DCEXS, Pompeu Fabra University (UPF). C/Dr. Aiguader, 88. 08003- Barcelona, Spain; 2 Departamento de Física, Facultad de Ciencias Exactas y Naturales, Universidad de Buenos Aires. Pabellón 1, Ciudad Universitaria, Buenos Aires, Argentina; 3 Instituto de Física de Buenos Aires, Consejo Nacional de Investigaciones Científicas y Técnicas. Pabellón 1, Ciudad Universitaria, Buenos Aires, Argentina; 4 Laboratorio de Biología de Sistemas Integrativa, Fundación Instituto Leloir, Buenos Aires, Argentina

## Abstract

Characterizing the behavior of disease genes in the context of biological networks has the potential to shed light on disease mechanisms, and to reveal both new candidate disease genes and therapeutic targets. Previous studies addressing the network properties of disease genes have produced contradictory results. Here we have explored the causes of these discrepancies and assessed the relationship between the network roles of disease genes and their tolerance to deleterious germline variants in human populations leveraging on: the abundance of interactome resources, a comprehensive catalog of disease genes and exome variation data. We found that the most salient network features of disease genes are driven by cancer genes and that genes related to different types of diseases play network roles whose centrality is inversely correlated to their tolerance to likely deleterious germline mutations. This proved to be a multiscale signature, including global, mesoscopic and local network centrality features. Cancer driver genes, the most sensitive to deleterious variants, occupy the most central positions, followed by dominant disease genes and then by recessive disease genes, which are tolerant to variants and isolated within their network modules.

With the application of next generation sequencing technologies to the identification of both germline and somatic variants across cohorts of patients as well as healthy individuals, the catalog of potential pathogenic variants is expanding rapidly. Recent findings have shown a large number of potentially damaging germline variants, but for most of them there is no information on the functional effect and its relation to disease^1^. Currently, our ability to interpret the effect of the variants discovered by genome sequence projects and how this leads to phenotypic variation and diseases is very limited. The fact that disease phenotypes are not caused by the individual action of genes but by their interaction in the context of biological networks further complicates the identification of clinically relevant variants. The discovery of somatic variants causing tumorigenesis is also a challenge. This field has advanced in recent years with the development of bioinformatics methods that detect signals of positive selection in genes across tumor samples, thus identifying the most likely driver candidates^2^,^3^,^4^. Nevertheless, uncovering driver mutations that occur at frequencies below the level of detection of these methods would require either sequencing even larger cohorts of tumors or new approaches incorporating prior knowledge of cancer genes features.

Biological networks are useful tools to model the complexity of the genotype-phenotype relation underpinning disease. In the last decade, protein-protein interaction networks (PINs) have extensively been exploited with the goal of unraveling the molecular mechanisms of a variety of human diseases^5^,^6^,^7^, and identifying novel disease genes candidates^8^,^9^,^10^. There is, thus, a body of scientific literature concerning the network properties of disease-related genes. According to these studies, disease genes possess distinctive topological properties that cannot be attributed solely to the fact that they have been extensively studied^11^,^12^,^13^. While cancer genes tend to occupy central positions in a PIN^6^, the scenario is less clear for other classes of disease genes. For example, Goh *et al*.^14^ found that if essential genes are excluded from the analysis of Mendelian disease genes, these do not show a tendency to occupy hub positions in the interactome^14^. Nevertheless, Xu and Li^12^ reported that Mendelian disease genes are more central in a literature curated PIN^12^. Later, Mendelian and complex disease genes were found to possess higher degrees and lower clustering coefficients than non-disease genes^13^. Currently it is not clear to what extent these contradictory results are caused by i) methodological issues, such as the sources of disease genes and/or PIN, ii) data incompleteness in any of these sources (disease genes, PIN), iii) discrepant topological definitions, or iv) actual variations in the network properties of genes belonging to different disease classifications^15^.

We have carried out a study to systematically determine whether and to what extent groups of genes resulting from different disease classifications –for example, clinical taxonomies vs. molecular genetics– possess distinct network properties. First, we have used a comprehensive catalog of disease genes, DisGeNET^16^, and culled different sets of genes corresponding to diverse disease classifications. Second, we carried out all our analyses across eight different PINs to investigate the impact of the selection of the network in the results. Third, we have analyzed local, mesoscale and global network properties of disease genes within the PIN. The analysis at the mesoscale level of the network provides insight into the modular organization of the PIN, potentially shedding light onto the mechanisms and regulation of cellular processes. Finally, we have also explored the relationship between the network properties of disease genes and their tolerance to likely deleterious germline variants. We were motivated by the lack of systematic studies addressing the question of whether the network location of different classes of disease genes correlates with their tolerance to possibly deleterious germline mutations. In probably the closest precedent, a recent study found that deleterious variants in the exomes of 1330 healthy individuals are located in the periphery of the PINs, while cancer somatic mutations appear in internal regions and monogenic disease variants are at intermediate network positions^17^.

We discuss our results in the context of a model that combines genetics (type of variation and mode of inheritance), genomic and interactomics (protein interactions through networks) to understand the mechanisms underlying human diseases.

## Results

In order to assess the network properties of different classes of disease genes, we obtained eight high quality PINs and analyzed them using an approach that combines topology and the modular structure of the network. Throughout the paper, we illustrate the results obtained with the HIPPIE protein interaction network^18^, but all analyses were replicated in other seven PINs. While six of the networks are aggregated interactomes from different studies which used different techniques, two networks were obtained from interactions derived uniquely from one experimental approach: a Yeast Two-Hybrid (Y2H) PIN and an Affinity Capture followed by Mass Spectroscopy (ACMS) PIN (See Methods and Supplementary Fig. S1 and Table S1).

The HIPPIE protein interaction network contains 9,580 proteins and 47,103 interactions. We found that 4,122 out of 7,412 disease genes, DGs (extracted from DisGeNET, see details in Methods) are included in HIPPIE (the number of genes mapping to other networks are in Supplementary Table S2). Notice that all PINs contain between 40–60% of DG, highlighting the incompleteness of our knowledge of the human disease interactome. Roughly half of the genes in the DG set are related to neoplasms (cancer related genes, CGs), while the other half are associated to other diseases (non-cancer disease genes or NCDGs). Out of the 4,122 DGs, 1,607 are related to Mendelian diseases (MGs), while 1,839 are related to complex diseases (CxDGs).

### Cancer genes are responsible for the network centrality of disease genes

We found that DG have a higher degree and betweenness than non-disease genes, but this trend is driven by cancer genes, as indicated by the decrease in the observed differences when CG are removed from the DG set (that even disappear in the case of the degree in 3 of the 6 PINs, Supplementary Figs S2 and S3). MGs and CxDGs have higher degree and betweenness than non-disease genes. The results for clustering coefficient are inconclusive (Supplementary Fig. S4). The behavior of this parameter seems to be linked to the nature of the method of detection of protein-protein interactions. Methods that capture indirect interactions, for example affinity purification with a bait protein, tend to produce more clustered networks, depending on the way that the interactions are annotated. This could lead to zones of artificially inflated estimates of clustering coefficients^19^.

Overall, these results provide systematic support to the observation made by various groups that cancer genes are more central to PINs, and present different local environments than NCDG^6^. Non-Cancer Disease Genes, on the other hand, do not show distinctive topological features, once the contribution of cancer genes is disregarded.

### The cartographic analysis of a PIN highlights mesoscale connectivity patterns

We next explored the connectivity of different groups of disease genes at the mesoscale level of the network –i.e., pertaining to its organization in clusters or modules. Despite the widespread use of network topological metrics like the betweenness or the degree of a node, it is worth noting that these features are not intended to explicitly mine mesoscale connectivity patterns. Betweenness related centrality indices may unveil interesting connectivity features at the global level, but they might not reflect them at a more local scale. Given that the degree of a node does not reflect the modular organization of the network, the use of degree-based centrality metrics might confer similar importance to genes that link different modules or are confined within a module. In addition, genes presenting low degree might be disregarded from a degree-centric point of view, even if they do play relevant connectivity roles in the biological network. Examples from HIPPIE include ADRBK2 (56^th^ and 43^rd^ percentile of degree and betweenness, respectively), a protein kinase involved in several signaling pathways, the phospholipase PLCB1 (48^th^ and 37^th^), that plays an important role in the intracellular transduction of many extracellular signals and in metabolism, the protease FURIN (48^th^ and 47^th^), and MAPK12 (61^st^ percentile for both), involved in pathways such as Signal Transduction (NGF, VEGF), Cell-Cell communication, Developmental Biology and Innate Immune System and Organelle biogenesis and maintenance.

**Figure 1:**
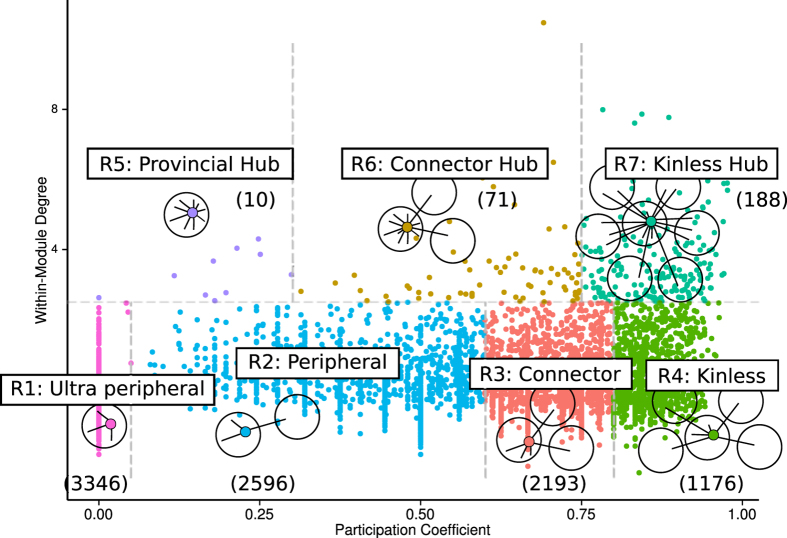
Cartographic partition of the nodes in the HIPPIE interactome. Description: The cartographic roles are represented in the z-P plane with different colors. In parenthesis, we show the number of genes in each role. Dashed lines in the figure delineate regions corresponding to the seven cartographic roles. We show a schematic representation of the type of connection for each role.

The mesoscale organization of the network is linked to its organization in clusters or modules. To unveil the modular organization of the PINs, we employed the *infomap* procedure^20^, one of the best performing network community recognition methodologies, which has produced sensible partitions of different types of complex networks^21^,^22^,^23^. After partitioning the PINs with *infomap*, we characterized the mesoscale connectivity features for each network node in terms of two parameters: the intra-cluster connectivity, z, and the participation coefficient, *P*^24^. The z parameter (equation (1)) standardizes the degree of a node in relation with the degree of nodes that belong to the same community, and the *P* parameter (equation (2)) quantifies the fraction of links that a given node projects to other communities (see Methods). We further categorized each network node according to the universal cartographic role classification scheme established by Guimerà and Amaral^24^,^25^. Figure 1 shows the distribution of HIPPIE nodes over the z-P plane. Dashed lines in the figure delimit regions corresponding to the seven cartographic roles^24^.

The classification of HIPPIE nodes into cartographic roles revealed a majority of poorly connected nodes playing peripheral (2,596) or ultra-peripheral (3,346) roles. More densely connected nodes were either connectors (2,193), with links more or less evenly distributed between the genes in their cluster and genes of other clusters, kinless nodes (1,176), displaying fewer than 35% of intra-cluster links, or kinless hubs (188) with more than half of their connections established with members of different clusters. The remaining nodes were either connector hubs (71), or provincial hubs (10). Reassuringly, we found that despite the differences in the PINs (Supplementary Fig. S1), the proportion of proteins in each cartographic role was very similar across all the eight studied PINs (Supplementary Table S3).

The cartographic analysis summarized relevant mesoscale interconnectivity features that might serve to highlight biologically sensitive patterns. For instance, the cartographic classification of the 6,608 proteins in HIPPIE annotated to Panther protein classes^26^ recapitulated major features of the architecture of cellular signaling pathways (Supplementary Fig. S5). Signaling molecules, membrane receptors and transporters were significantly enriched for ultra-peripheral and peripheral HIPPIE nodes. Furthermore, nodes in these cartographic roles exhibited a clear enrichment for Gene Ontology terms related to the activity of membrane receptors –many are ligands, receptors or receptor modulators– and transporters (Supplementary Table S4). Conversely, proteins with high participation roles (kinless and kinless hubs) were most significantly enriched for chaperone functioning and regulatory classes, such as kinase, ligase, transferase and nucleic acid binding activities from Panther (Supplementary Fig. S5), and chromatin maintenance, regulation of transcription and regulation of ubiquitin mediated proteolysis from the Gene Ontology molecular function (Supplementary Table S4).

### Different types of disease genes show distinctive connectivity patterns at the mesoscale level

We analyzed the mesoscale features of disease genes by looking at the overrepresentation of each set of disease genes across the seven network cartographic roles (Fisher exact Test, Table 1) for the different gene sets analyzed. Disease genes as a group (see DG column, Table 1) exhibited significant enrichment for connector, kinless and kinless hub roles. However, this signal disappears when CGs are removed from the set of DGs (see NCDGs column, Table 1). CGs are enriched for nodes with high participation roles, such as kinless (p-value in the order of 10^−38^), kinless hub (p-value in the order of 10^−10^) and connector (p-value in the order of 10^−9^). This result is consistent across the six PINs (Supplementary Fig. S6). NCDGs are homogeneously distributed amongst roles, which underlines that CGs, again, are solely responsible for the observed enrichment of DGs for high participation roles.

The fact that cancer genes have high degree values in PINs could partially explain the observed enrichment for the kinless-hub role, given the existent role-to-degree relationship (Supplementary Fig. S7). However, the enrichment for non-hub roles (connector and kinless roles) revealed a qualitatively different participation-based bias, more directly linked to inter-modular connectivity patterns. Moreover, the fact that the enrichment of CGs for kinless nodes was several fold greater than their enrichment for nodes in connector roles suggests that cancer genes tend to connect many separate modules of the network, rather than genes in their close vicinity which belong to the same cluster. To further validate this hypothesis we decided to de-convolve the degree signal from the enrichment results observed for CG genes performing a degree-aware bootstrap test for the cartographic role enrichment calculation (see Methods). Interestingly, we found that connector (p-value < 10^−3^) and kinless (p-value < 10^−3^), but not the kinless hub (p-value = 0.92) category enrichment remain significant under the bootstrap analysis. These results suggest that CG genes display a non-trivial enrichment for non-hub, high participation cartographic roles, which cannot be explained by the effect of the degree distribution, but is cemented on mesoscale connectivity patterns.

When assessing the difference between genes related to complex and Mendelian diseases, we found that CxDGs are overrepresented amongst kinless and kinless hub genes, whereas MGs are enriched for kinless and connector genes. However, both trends disappeared after removing the CGs from each gene set (Table 1). We observed a similar behavior when genes are grouped according to MeSH disease classes. With the exception of Parasitic and Eye diseases, the corresponding gene sets are similarly enriched for kinless and kinless hub nodes (Supplementary Fig. S8B).

Nevertheless, this trend disappears when CG are removed from each of the MeSH disease genes. We reasoned that other disease categorizations, more homogeneous in terms of genetic or molecular mechanisms might result in gene sets with clearer network trends. In order to further investigate this hypothesis, we subdivided MGs according to their inheritance mode into autosomal dominant (AD) and autosomal recessive (AR) disease genes. In addition, we filtered the set of cancer related genes (CGs) to keep only genes related to tumorigenesis upon somatic alterations (drivers). The mapping of the gene sets into the different PINs is summarized in Supplementary Table S2. There is a certain degree of overlap between these sets because some genes may contribute to different diseases. For instance, some germline variants in several well-known loss of function cancer driver genes such as DNM2, SMAD4, NF1, PTCH1, PTEN, SMARCB1, TSC1, cause dominant negative Mendelian diseases^27^.

While AD genes are enriched for kinless and kinless hub roles (Table 2), AR genes are not significantly overrepresented within any role in HIPPIE. Nevertheless, they do exhibit enrichment for ultra-peripheral nodes, in two of the PINs (BioGRID, p-value in the order of 10^−2^ and IntAct p-value in the order of 10^−3^, Supplementary Fig. S9). Driver genes show significant enrichment for kinless and kinless hub roles (Table 2). Removing the driver genes from AR and AD sets resulted in a decrease of the enrichment for nodes of high participation roles for AD genes and, inversely, an increase of the enrichment of the AR genes for nodes of low participation roles. Noticeably, the enrichment of drivers (p-value = 0.001), AD (p-value = 0.001), and AD non-driver genes (p-value = 0.026) for kinless nodes remains significant under the degree-aware bootstrap analysis. This last result stresses that these gene sets display non-trivial connectivity patterns at the network mesoscale level. The network parameters of groups of disease genes computed from the Y2H and ACMS PINs behave similarly to other networks. Cancer drivers and AD genes are enriched for high participation roles (kinless, and kinless hub, and connector in the case of drivers in ACMS PIN), while AR genes do not play a preferential role (Supplementary Fig. S10).

### The tolerance of different types of disease genes to likely deleterious germline variants reflects the heterogeneity of their network roles

We hypothesized that disease genes with higher-than-average participation in the network must be under strong purifying selection and therefore, be less tolerant to likely deleterious variants across human populations. To the best of our knowledge, there is no previous study that systematically addresses the relationship between the tolerance to germline mutations with the network properties of disease genes. Therefore, we asked next whether genes involved in diseases of different classes, which display distinctive network roles, exhibit different sensitivity to likely deleterious germline variants. To answer this question, we retrieved the germline variants detected across 60,706 exomes (ExAC) and kept those falling into one of two groups. In the first group, we included protein sequence affecting variants –missense, stop gained, stop lost, frameshift, splice donor and splice acceptor variants– with CADD score > 15 and considered them as likely deleterious variants^28^. The second group comprised synonymous variants, which were considered non-deleterious. We then computed, for each gene, a High-impact-to-Synonymous variants Ratio (HS Ratio) as the quotient between the number of likely deleterious variants and the number of non-deleterious variants (see Methods). We use this HS Ratio as a proxy of the sensitivity of genes to likely deleterious germline variants.

Cancer drivers and AD genes exhibit lower HS Ratio than the average genes (Table 3), denoting a higher-than-average sensitivity to likely deleterious variants in human populations. The trend of AD genes towards lower-than-average HS Ratio becomes less significant in the AD_ND_ set. AR genes, on the other hand, show significant less sensitivity to such deleterious variants than average genes in the PINs.

**Figure 2:**
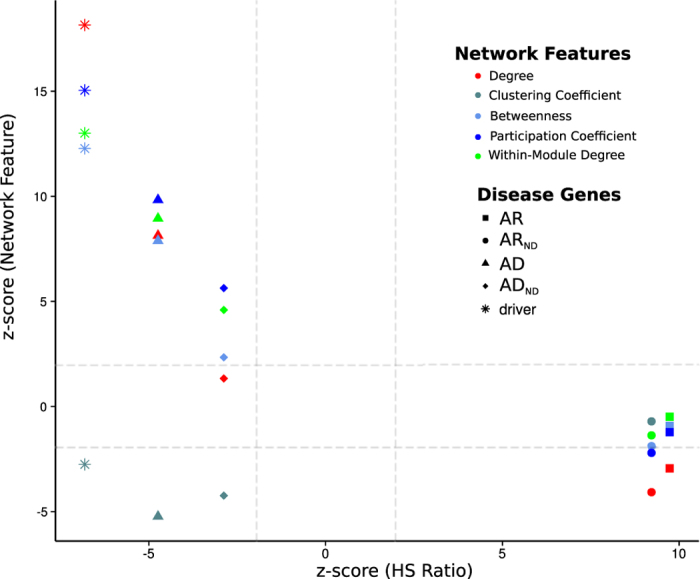
Relationship between network features and the HS Ratio for the disease gene sets in HIPPIE. Description: We plot the z-scores resulting from 10,000 randomizations of each network feature (degree, betweenness, clustering coefficient, participation coefficient, and within-module degree) and of the HS Ratio for each set of disease genes (AD, AD_ND_, AR, AR_ND_, driver) in HIPPIE. AD: Autosomal Dominant, AD_ND_: AD genes without driver genes, AR: Autosomal Recessive, AR_ND_: AR genes without driver genes, driver: cancer driver genes.

Figure 2 illustrates the double separation that takes place between groups of disease genes and non-disease genes in terms of network features and the tolerance to likely deleterious germline variants. We have plotted the z-score of each parameter resulting from 10000 randomizations for each set of genes (see Methods). It can be observed from the figure that the more significantly lower the HS Ratio for a given disease gene set, the more significantly higher the corresponding centrality indices. Differences in network centrality metrics were particularly large for drivers and AD genes. A moderate bias toward high values of mesoscale centrality features could still be recognized for the set of non-driver AD genes (AD_ND_ set). AR genes show almost no differences in their network features with respect to the average gene in the network across all PINs but exhibit significantly-higher-than-average HS Ratio. They do exhibit smaller degree (in 4 out of 8 PINs, Supplementary Figs S11 and S12) and smaller participation coefficient than the average node in the BioGRID and IntAct PINs (Supplementary Figs S11 and S12). Again, this trend became stronger when known driver genes were removed from the AR set, so that it became significant for HIPPIE as well (Supplementary Fig. S11). In summary this analysis uncovers a relationship between a gene’s centrality in the PIN and its sensitivity to likely deleterious germline variants. Noticeably this proved to be a network multiscale signature, as the same trend is observed when global (i.e. betweenness), mesoscopic (within-module degree or participation coefficient) and local (degree) network centrality features are considered.

**Figure 3:**
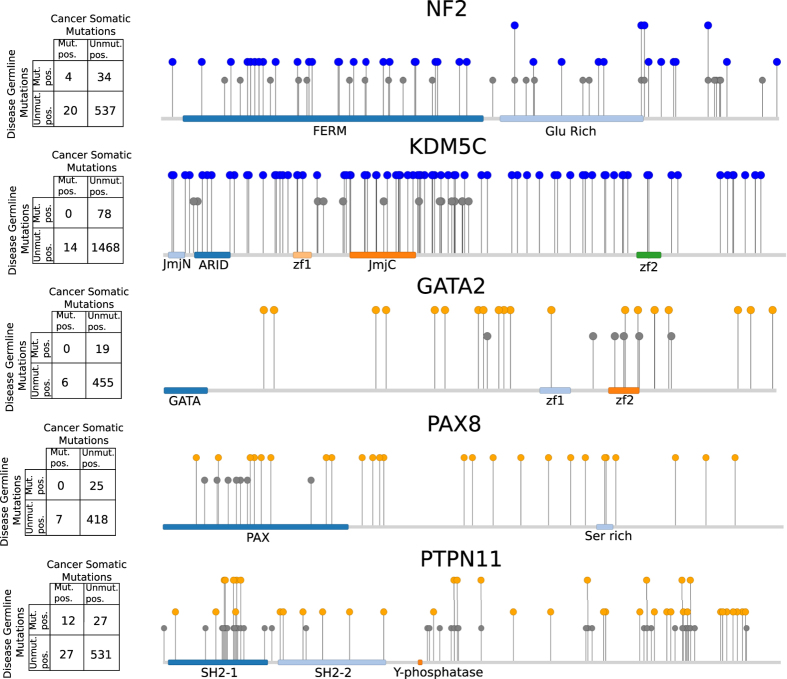
Distribution of disease-associated germline and cancer somatic mutations in genes NF2, KDM5C, GATA2, PAX8, and PTPN11. Description: We show examples of the positions of germline and somatic mutations for five cancer driver genes also associated to Mendelian diseases with different inheritance mode. In panel A, the contingency table of the Fisher test is shown. Mut pos: mutated position, Unmut pos: position where there are not annotated mutations. In panel B, a schematic representation of the position of the germline and somatic mutations is shown.

Finally, we focused on the subset of genes that are both cancer drivers and associated to Mendelian diseases. These genes behave collectively –in terms of centrality, molecular activities and sensitivity to probably deleterious germline variants– like driver genes. We hypothesized that the deleterious germline variants in these genes that cause Mendelian disorders affect positions in the protein sequence that are different from those affected by somatic mutations that turn the gene into a cancer driver –because changes at these positions would likely result in lethal phenotypes. We tested this hypothesis on a group of 81 driver genes (35 loss-of-function and 46 gain-of-function drivers) on which deleterious germline variants causing a Mendelian disorder have been mapped to at least three separate positions. Specifically, we asked whether somatic mutations with tumorigenic potential (non-synonymous mutations on oncogenes and non-synonymous and truncating mutations in tumor suppressors) and disease related germline variants tend to occur at different positions. We found that this is the case for the majority of these 81 genes −65 of them possess Fisher’s odds-ratios below 0.1 (Supplementary Table S5). In Fig. 3 we show examples of loss-of-function (NF2 and KDM5C) and gain-of-function (GATA2, PAX8, and PTPN11) driver genes with little or no overlap between germline and somatic mutations. Exceptions to this trend are genes that suffer germline variants that confer susceptibility to cancer, such as the von Hippel-Lindau syndrome caused by some variants in VHL; Cowden disease 5 caused by mutations in PIK3CA; Li-Fraumeni syndrome 1 and TP53; and proteins related to the RAS family, or belonging to RAS pathways, whose germline mutations produce developmental diseases that frequently increase the risk of cancer^29^.

## Discussion

Our results show that the network centrality of different classes of disease genes, including complex, Mendelian and clinical-oriented classifications, is mostly attributable to cancer genes. Cancer genes are central not only in terms of number of neighbors, but also in terms of the clusters they connect. Remarkably, we found that high Participation roles played by cancer genes are not explained by their higher number of interactions, but by their unique inter-modular connectivity patterns. We also found that these connectivity patterns differ for disease genes with contrasting inheritance modes: while autosomal dominant genes play high participation roles, autosomal recessive genes are more confined to their own modules. Interestingly, the network roles of these two different types of disease genes relate to their tolerance to likely deleterious germline variants: the more central the disease genes are, the more sensitive to damaging germline variants. Our findings may explain some of the seemingly contradictory results reported so far^12^,^13^,^14^, which found that “disease”, “complex disease”, and “Mendelian disease” genes occupy central network positions, but are probably measuring the centrality of cancer genes. Our study found that only autosomal recessive Mendelian genes are overrepresented in the periphery of the network, contrasting previous reports^14^. Additionally, we found that autosomal dominant Mendelian genes possess network properties that are in part driven by a small subset of driver genes overlapping with them. This also might explain part of the differences between the results of previous studies, which have largely ignored that some genes linked to dominant and recessive Mendelian diseases are also cancer drivers. In summary, our results put in perspective previous observations regarding the properties of disease genes as a whole and even question the rationale behind the analysis of such heterogeneous sets of disease genes.

Our findings are reproducible across six different PINs compiled from six different resources. Additionally, to minimize the any reporting biases in these resources, we complemented our analysis with two PINs derived from single experimental methodologies and found consistent results. This reproducibility supports the robustness of our main conclusions against any bias or inherent-incompleteness of a particular protein-protein interaction dataset^30^.

We found compelling evidence both from topological and cartographic analysis of PINs that among all disease related genes, cancer drivers occupy the most central roles, significantly expanding a previous report focused on 21 Chronic Lymphocytic Leukemia drivers^17^. In particular, the cartographic analysis showed that cancer drivers are very significantly overrepresented among the proteins that connect several modules of the PIN (kinless and kinless hub nodes). Genes that play these roles are frequently involved in very core cellular processes, such as signal transduction through several pathways, the regulation of transcription, chromatin maintenance, and ubiquitin mediated proteolysis. The enrichment of driver genes for these two central roles also explains why they are significantly more sensitive to likely deleterious germline variants. Probably an important fraction of deleterious germline variants affecting these genes are filtered out by purifying selection while somatic mutations that affect them have a high likelihood of causing tumorigenesis probably because they impact on key cellular functions^31^,^32^,^33^. A fraction of driver genes, nevertheless bear deleterious variants that are not lethal, but cause Mendelian diseases. Interestingly, the majority of the driver genes that play a role in Mendelian disease have a dominant inheritance mode. This might explain why autosomal dominant genes resemble driver genes. Some of these genes increase predisposition to cancer, for example, in rasopathies, and BRAF, KRAS, HRAS, NF1, NRAS PTPN11, RAF1^29^. These genes behave like drivers both in the roles they play in the PIN and in their sensitivity to likely deleterious variants, rather than as Mendelian disease genes. In summary, our findings suggest that if a gene plays high participation roles in the network, deleterious germline variants affecting it will have high probability to be filtered out by purifying selection, while somatic mutations impacting its function, will cause tumorigenesis with high probability.

Autosomal recessive genes show a behavior entirely opposite to that of cancer drivers. They tend to be less central (as observed by^34^,^35^), enclosed within a network module and exhibiting low or null participation and low degree, and they are significantly more tolerant to likely deleterious variants than other genes in the network. The effect of variants in one such gene would be confined to its own module. Autosomal dominant genes occupy an intermediate position between autosomal recessive and drivers. Proportionally, they represent a smaller share of kinless and kinless hubs than drivers; and coherently, they are less sensitive to likely deleterious germline variants, although still more than the average gene in the interactome.

Deleterious variants in genes associated to dominant diseases might be dominant negative or produce haploinsufficiency^36^,^37^ resulting in the disease phenotype. In the first case, the protein product of a mutated allele ultimately produces an aberrant protein complex, whereas in the second the protein level produced by the normal allele is not sufficient to fulfill the entire functionality of the protein. The former mechanism may fit better the behavior of Autosomal dominant genes encoding structural proteins, which are enriched for non-truncating variants, while the latter may explain better the case of transcription factors, enriched for truncating variants^7^. These two mechanisms could explain why some loss-of-function drivers, such as TP53, PTEN, RB1, and APC may also behave as Mendelian dominant genes. In the first case, while both alleles of the gene may be rendered inactive by alterations in cancer, deleterious germline variants in only one allele may produce a defective copy of the protein which in turn produces a faulty multimer composed of both active and inactive subunits of the protein. As for haploinsufficiency, the decrease in the level of active protein caused by a deleterious germline variant on one allele of the gene, determines the disruption of at least some of the functions –maybe by the failure to fulfill all its interactions, or to maintain signaling through certain pathways at homeostatic levels– carried out by the protein. In the haploinsufficiency scenario, the more complexes a protein is involved in, the more likely it is that a decrease of its level results in disease.

To sum up, autosomal recessive diseases result from homozygous mutations of genes highly tolerant to deleterious variants, involved in very few interactions, confined to their own pathways or modules. They produce perturbations that are equivalent to removing a network node^7^. While node removal of Autosomal dominant genes is probably lethal to the cell, variants affecting only one allele, involving one or a few of their many interactions –i.e, edge perturbations— might trigger Autosomal dominant diseases. Most cancer drivers would be intolerant to these edge perturbation events, probably because they are incompatible with the development of a viable organism. Nevertheless, somatic cells may acquire growth advantages and eventually become malignant from mutations that cause either edge perturbations (new interactions, or their hyper activation, in the case of oncogenes) or node removal (tumor suppressors) of cancer driver genes.

Despite the incompleteness of our current knowledge on disease genes and the human interactome, network science has been shown to be a valuable tool to study human diseases^30^. Computational methods that attempt to prioritize candidate disease genes –from exome sequencing data or exploiting the guilty-by-association principle– or to identify driver genes from somatic mutations across cohorts of tumors could benefit from our knowledge to improve their performance. For instance, the aforementioned differences in the sensitivity of driver, Autosomal dominant and Autosomal recessive genes to likely deleterious germline variants may refine approaches like the one proposed by Petrovski *et al*.^38^, based on the residual variation intolerance score (RVIS) to quantify gene intolerance to functional mutations, genome-wide, or Shyr *et al*.^39^, that ranked genes based on their frequency of rare non-synonymous/splice-site variants in general populations. Our results can also be applied to a scoring system of gene-disease associations inferred through text mining. For example, such a scoring system would give weights to genes taking into account their classification into cartographic roles.

## Methods

### Assembling the Protein-protein Interaction Networks

Protein interaction data were retrieved from HIPPIE^18^, BIANA^40^, BioGRID^41^, IntAct^42^, IrefIndex^43^ and from Human Interactome Project (HBI)^19^,^44^. All files were obtained in December 2014. We built 6 PINs, one for each resource. Additionally, we created two networks derived from single experimental methods: a Yeast Two-Hybrid (Y2H) PIN and an affinity capture followed by mass spectroscopy (ACMS) PIN. Notice that interactions predicted through computational approaches were not included in any of the networks used in this study.

The source, number of genes and set of interactions in each PIN, as well as the overlaps between them are shown in Supplementary Fig. S1. The giant component of the six PINs contained 7,000–12,000 proteins, and 25,000–70,000 interactions after filtering to retain only high-confidence interactions^45^. The overlap between pairs of PINs (Jaccard’s index) ranged between 0.5–0.7 for genes and 0.1–0.35 for interactions. Similarly small overlaps have been reported before^44^,^46^,^47^,^48^, and are mainly attributed to the complementary nature of different protein interactions detection methods^48^ and study bias. Throughout all the paper we illustrate the results with the HIPPIE PIN, and we show the results for the rest of the networks as Supplementary Material.

### HIPPIE

We used only the interactions with a score greater than, or equal to 0.72, corresponding to the 25% of the highest scoring interactions, as suggested by the authors^18^. The file was downloaded from http://cbdm.mdcberlin.de/tools/hippie/index.php, v1.7.

### BIANA

We did not include interactions obtained by methods producing co-complexes. The data was obtained from http://sbi.imim.es/web/index.php/research/servers/biana.

### IntAct

We only kept interactions annotated as human. Using the same quality criteria as in HIPPIE, we kept the 25% of the top scoring interactions. The data was obtained from ftp://ftp.ebi.ac.uk/pub/databases/intact/current/psimitab/intact.zip.

### Human Binary Interactome (HBI)

We merged the files, HI-I-05, HI-II-14, Lit-BM-13, downloaded from http://interactome.dfci.harvard.edu/H_sapiens/index.php.

### iRefIndex

We only kept interactions annotated as human, and detected by more than one method. The data was obtained from http://irefindex.org/download/irefindex/data/archive/release_13.0/psi_mitab/MITAB2.6/9606.mitab.08122013.txt.zip.

### BioGRID

We only kept interactions annotated as human, and reported by at least one experimental system, or at least two different publications. The data was obtained from http://thebiogrid.org/downloads/archives/Release%20Archive/BIOGRID-3.2.120/BIOGRID-ALL-3.2.120.tab2.zip.

### Yeast Two-Hybrid

From each resource, we kept interactions obtained by yeast two-hybrid approaches.

### Affinity Capture/Mass Spectrometry

From HIPPIE and BioGRID, we obtained the interactions annotated as “Affinity Capture-MS” (the rest of the resources annotations do not provide this level of detail).

For all PINs (except HBI), we removed interactions containing genes UBC, SUMO1, CUL1, COPS5, SUMO2, CUL3, NEDD8, SUMO3, RNF2, UBD, CUL7, CAND1, SIRT7 FN1, OBSL1, FN1, FBXO6, and CCDC8, following the criteria in^44^.

For each PIN, we show the average of different network parameters in Supplementary Table S1. In addition, we characterize each network according to *study bias* and *scale of experimental approach* used to detect the interactions. The interactome networks can be classified based on the experimental methods used to detect the interactions, but also based on the focus of the original study. For example, a study on protein-protein interactions of kinases will be highly *biased* towards this type of proteins, even if the study uses an Y2H approach to detect the interactions. Therefore, we regard an *unbiased study* as one aimed at exploring the whole proteome to detect protein-protein interactions. In this scenario it will make sense to apply high throughput approaches for practical reasons. On the other hand, in a *study biased* towards a particular class of proteins (e.g. kinases, cancer genes, etc.) both low throughput and high throughput techniques could be applied.

In order to determine if a particular interaction between proteins comes from an *unbiased* study, we determined if the interaction has been reported in any of the following papers: Rual *et al*.^19^; Stelzl *et al*.^49^; Ewing *et al*.^50^, Venkatesan *et al*.^51^; Wang *et al*.^52^; Kristensen *et al*.^53^, Havugimana, P. C. *et al*.^54^; and Rolland, T. *et al*.^44^. We consider that these studies are unbiased because they do not interrogate specific portions of the interactome, but aim at characterizing the full proteome of a cell/organism. Other studies exploring specific sections of the human interactome, such as the ubiquitin-related or signal transduction processes^55^,^56^,^57^,^58^,^59^, are therefore considered *biased*.

The other factor that can be taken into account is the scale of the experimental approach used to detect the interactions. We classify interactions according to the scale of the experimental method in two classes: a) High-throughput: interactions from a study reporting more than 100 interactions, b) Low-throughput: interactions from a study reporting fewer than 100 interactions.

A similar approach is employed to classify interactions by BioGRID^41^. With this information we assessed the percentage of unbiased, high-throughput scale, and low-throughput interactions contained in each PIN (Supplementary Table S1).

In addition, we also provide in the Supplementary Table S2 a characterization of the 8 networks in terms of the coverage of each class of disease genes used in this study.

### Mapping disease genes to the networks

#### Disease Genes

We used DisGeNET ( http://disgenet.org, version 2.1, 5/5/2014), as source of disease genes^16^. We filter the disease phenotypes contained in DisGeNET using their UMLS® Metathesaurus® semantic type and their MeSH Class. We kept only semantic types T019, T047, T048, and T191 corresponding to Congenital Abnormality, Disease or Syndrome, Mental or Behavioral Dysfunction, and Neoplastic Process, respectively, and disease classes from C01 to C20, C25, F01 and F03 (see Supplementary Fig. S8 for details of the MeSH disease classes). We restricted the disease genes to those reported by CURATED sources^16^. The disease genes were further classified in the following categories:

#### Cancer-related genes

Genes annotated to diseases classified as Neoplasms in DisGeNET. The involvement of these genes in neoplastic diseases varies: they might be driver genes, or may have been reported as altered in some tumor type and behave as passengers.

#### Cancer Driver Genes

We downloaded Cancer Genes Census genes on August 25, 2014^60^. A second driver list of 464 genes was obtained from^61^. After merging both lists, there were 781 driver genes.

#### Mendelian disease genes and inheritance modes

Mendelian disease genes were retrieved from OMIM^62^ on August, 2013. We excluded genes with associations to disease marked as susceptibility. Inheritance modes of the genes were obtained from two datasets^63^,^64^, who manually curated inheritance information from OMIM. We removed the genes with contradictory annotations. We obtained 1,153 AR genes and 954 AD genes.

#### Complex Disease genes

We manually compiled a list of complex diseases, using DisGeNET diseases (CURATED). From the Disease Genes List, we excluded Bacterial Infections and Mycoses (C01), Virus Diseases (C02), Parasitic Diseases (C03), Neoplasms (C04) MeSH disease classes, terms corresponding to broad disease classes (such as Brain diseases, Kidney diseases, Autoimmune diseases), phenotypes, signs and symptoms, and Mendelian diseases. At the end, we obtained 2,863 genes associated to 644 complex diseases.

### Mutation rate and Functional Impact assessment

Germline variants were detected across 60,706 exomes, with the data downloaded from Exome Aggregation Consortium (ExAC), Cambridge, MA (URL: http://exac.broadinstitute.org) [accessed November, 2014]. We used Gencode annotations (ENSEMBL) to find canonical transcripts and a deleteriousness score was computed using Combined Annotation Dependent Depletion (CADD) scores^28^ ( http://cadd.gs.washington.edu/). Extremely rare variants (less than 10^−5^) were excluded from the analysis. Only mutations in coding regions, of type synonymous, non-synonymous, splicing site, stop gain and stop lose and frameshift were analyzed. A CADD (scaled) score of more than 15 was used to classify a variant as high impacting. We then calculated the High-impact-to-Synonymous variants Ratio (HS Ratio) as the quotient between the number of likely deleterious variants and the number of non-deleterious variants for all genes (17,438 genes). We did not include in the analysis genes with less than four synonym or protein sequence affecting variants (missense, stop gained, stop lost, frameshift, splice donor and splice acceptor variant).(CADD) scores^28^ ( http://cadd.gs.washington.edu/). Extremely rare variants (less than 10^−5^) were excluded from the analysis. Only mutations in coding regions, of type synonymous, non-synonymous, splicing site, stop gain and stop lose and frameshift were analyzed. A CADD (scaled) score of more than 15 was used to classify a variant as high impacting. We then calculated the High-impact-to-Synonymous variants Ratio (HS Ratio) as the quotient between the number of likely deleterious variants and the number of non-deleterious variants for all genes (17,438 genes). We did not include in the analysis genes with less than four synonym or protein sequence affecting variants (missense, stop gained, stop lost, frameshift, splice donor and splice acceptor variant).

### Positional Analysis of Mutations

We obtained the deleterious variants associated to disease from the Human polymorphisms and disease mutations file ( http://www.uniprot.org/docs/humsavar, release 2015_01 of 07-Jan-2015) from UniProt^65^ and from ClinVar file ftp://ftp.ncbi.nlm.nih.gov/pub/clinvar/tab_delimited/variant_summary.txt.gz downloaded on January, 2015. We kept variants annotated as “disease” in UniProt and as “Pathogenic” in ClinVar^66^. We obtained cancer mutations from^61^. We kept genes where at least three mutations of both types were found. We obtained the length of the coding sequence from UniProt.

### Network Clustering

In order to assign roles to the genes, we partitioned the PIN in clusters using *infomap* algorithm^20^, using the implementation available at: http://www.tp.umu.se/~rosvall/code.html.

### Cartography

According to Guimerà and Amaral^24^ the genes were assigned to one of the following roles: ultra-peripheral nodes, peripheral, non-hub connector, non-hub kinless, provincial hubs, connector hubs, kinless hubs. These seven different roles are heuristically defined, using their localization in the different regions of the z–P parameter space, where z (within-module degree) and P (participation coefficient) are calculated according to equations (1) and (2), respectively.


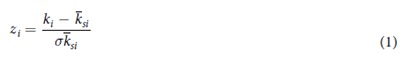


where k_i_ is the number of links of node i to other nodes in its module, 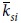 is the mean degree of all nodes in cluster s_i_, and 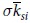 is the standard deviation of the degree in the cluster s_i_


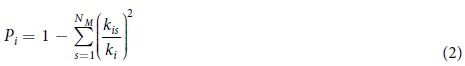


where k_is_ is the number of links of node i to nodes in the module s, and k_i_ is the total degree of node i.

Nodes with z > 2.5 are classified as module hubs and nodes with z < 2.5 as non-hubs. Both hub and non-hub nodes are then further characterized by using their participation coefficient.

Non-hub nodes can be divided into four different roles: (R1) ultra-peripheral nodes; that is, nodes with all their links within their module (P ≤ 0.05); (R2) peripheral nodes; that is, nodes with most links within their module (0.05 < P ≤ 0.62); (R3) non-hub connector nodes; that is, nodes with many links to other modules (0.62 < P ≤ 0.80); and (R4) non-hub kinless nodes; that is, nodes with links homogeneously distributed among all modules (P > 0.80). Similarly, hub nodes are assigned to: (R5) provincial hubs; that is, hub nodes with the vast majority of links within their module (P ≤ 0.30); (R6) connector hubs; that is, hubs with many links to most of the other modules (0.30 < P ≤ 0.75); and (R7) kinless hubs; that is, hubs with links homogeneously distributed among all modules (P > 0.75).

### Panther Database

We downloaded the file containing family/subfamily name, and the molecular function, biological process, and pathway classifications corresponding to Release 9.0^67^ ftp://ftp.pantherdb.org//hmm_classifications/current_release/PANTHER9.0_HMM_classifications and mapped the UniProt identifiers to Entrez gene identifiers.

### Network and Statistical Analysis

The network analysis was carried out using R (version 3.1.0)^68^ and the iGraph Library (version igraph_0.7.1)^69^. We used package Gostats_2.30.0^70^ to perform the Molecular Function GO enrichment analysis. Other statistical test, such as Fisher and Mann Whitney U test were also performed in R. All multiple testings were corrected using Benjamini & Hochberg method.

### Degree control for role enrichment estimation

A bootstrapping procedure was devised to control the node’s degree distribution confounding factor for the role enrichment analysis. For each enrichment test we considered an ensemble of 1,000 control random gene-sets having the same degree distribution than the genes under study (we disregarded from the analysis the top 5% of nodes presenting the highest degree values, i.e. k > 50). A p-value level was assigned according to the number of random realizations displaying the same or larger effects (over/under representation significance) than the ones observed in the original data. Each random realization was built blindly selecting genes from pools of given degree levels in order to follow the degree distribution displayed by the original gene set.

### Statistical significance of gene sets features

For each gene set (AD, AD_ND_, AR, AR_ND_ and driver), we generated 10,000 randomly selected samples of genes from the network of the same size of the gene set. Then, we computed the mean value of each sampled feature (degree, betweenness, clustering coefficient, participation coefficient, within-module degree, and HS Ratio) for the 10,000 randomizations. From this distribution of means a z-score was calculated for every gene set and feature pair.

## Additional Information

**How to cite this article**: Piñero, J. *et al*. Uncovering disease mechanisms through network biology in the era of Next Generation Sequencing. *Sci. Rep.* **6**, 24570; doi: 10.1038/srep24570 (2016).

## Supplementary Material

Supplementary Information

## Figures and Tables

**Table 1:** Overrepresentation of each cartographic role across disease gene sets in the HIPPIE interactome.

Cartographic role	DG	CG	NCDG	CxDG	CxDG_NC_	MG	MG_NC_
kinless hub	1,84E-07	3,75E-10	1	1,82E-09	0,175	0,122	1
connector hub	0,100	0,137	0,570	0,002	0,122	1	1
provincial hub	0,076	0,134	0,627	1	1	0,065	0,562
kinless	2,41E-26	2,55E-38*	1	1,76E-10	1	1,84E-07	1
connector	3,41E-06	1,82E-09*	1	0,142	1	0,016	0,627
peripheral	1	1	0,562	1	0,172	1	1
ultra-peripheral	1	1	0,562	1	1	1	0,122

Legend: DG: all disease genes, NCDG: non-cancer disease genes, CG: cancer genes, CxDG: complex disease genes, CxDG_NG_: CxDG without cancer genes, MG: Mendelian disease genes, MG_NG_: MG without cancer genes. We show the p-values of the Fisher test, corrected by multiple testing according to Benjamini & Hochberg method.

^*^Set of genes that remain significant under the bootstrap analysis.

**Table 2:** Overrepresentation of each cartographic role across disease gene sets in the HIPPIE interactome.

Cartographic role	AD	AD_ND_	AR	AR_ND_	driver
kinless hub	3,51E-05	0,012	1	1	3,51E-05
connector hub	1	1	1	1	0,003
provincial hub	0,103	1	0,343	0,329	0,097
kinless	1,32E-10*	0,002*	0,868	1	4,15E-28*
connector	0,015	0,103	0,932	0,932	0,005
peripheral	1	1	1	1	1
ultra-peripheral	1	1	0,281	0,063	1

Legend: AD: Autosomal Dominant, AD_ND_: AD genes without driver genes, AR: Autosomal Recessive, AR_ND_: AR genes without driver genes, driver: cancer driver genes. We show the p-values of the Fisher test, corrected by multiple testing according to Benjamini & Hochberg method.

^*^Set of genes that remain significant under the bootstrap analysis.

**Table 3:** Average high-impact to synonymous ratio (HS Ratio) of the disease gene sets.

Disease gene set	N	HS Ratio	z-score	p-value	Corrected p-value
driver	691	0,759	−6.881	5,93E-12	9,89E-12
AD	750	0,809	−4.720	2,36E-06	2,95E-06
AD_ND_	589	0,838	−2.462	1,38E-02	1,38E-02
AR	684	1.108	9.690	3,34E-22	1,67E-21
AR_ND_	641	1.112	8.754	2,06E-18	5,15E-18

Legend: AD: Autosomal Dominant, AD_ND_: AD genes without driver genes, AR: Autosomal Recessive, AR_ND_: AR genes without driver genes, driver: cancer driver genes. N is the number of genes in each set that maps to HIPPIE, the z-score is the result of 10,000 randomizations, and the p-values are computed from the z-score.

## References

[b1] Durbin R. M. . A map of human genome variation from population-scale sequencing. Nature 467, 1061–1073 (2010). 20981092 10.1038/nature09534 PMC3042601

[b2] Lawrence M. S. . Discovery and saturation analysis of cancer genes across 21 tumour types. Nature 505, 495–501 (2014). 24390350 10.1038/nature12912 PMC4048962

[b3] Tamborero D. . Comprehensive identification of mutational cancer driver genes across 12 tumor types. Sci. Rep. 3, 2650 (2013). 24084849 10.1038/srep02650 PMC3788361

[b4] Kandoth C. . Mutational landscape and significance across 12 major cancer types. Nature 502, 333–9 (2013). 24132290 10.1038/nature12634 PMC3927368

[b5] Chuang H.-Y., Lee E., Liu Y.-T., Lee D. & Ideker T. Network-based classification of breast cancer metastasis. Mol. Syst. Biol. 3, 140 (2007). 17940530 10.1038/msb4100180 PMC2063581

[b6] Jonsson P. F. & Bates P. A. Global topological features of cancer proteins in the human interactome. Bioinformatics 22, 2291–7 (2006). 16844706 10.1093/bioinformatics/btl390 PMC1865486

[b7] Zhong Q. . Edgetic perturbation models of human inherited disorders. Mol. Syst. Biol. 5, 321 (2009). 19888216 10.1038/msb.2009.80 PMC2795474

[b8] Köhler S., Bauer S., Horn D. & Robinson P. N. Walking the interactome for prioritization of candidate disease genes. Am. J. Hum. Genet. 82, 949–58 (2008). 18371930 10.1016/j.ajhg.2008.02.013 PMC2427257

[b9] Lage K. . A human phenome-interactome network of protein complexes implicated in genetic disorders. Nat. Biotechnol. 25, 309–16 (2007). 17344885 10.1038/nbt1295

[b10] Guney E. & Oliva B. Exploiting protein-protein interaction networks for genome-wide disease-gene prioritization. Plos One 7, e43557 (2012). 23028459 10.1371/journal.pone.0043557 PMC3448640

[b11] Lim J. . A protein-protein interaction network for human inherited ataxias and disorders of Purkinje cell degeneration. Cell 125, 801–14 (2006). 16713569 10.1016/j.cell.2006.03.032

[b12] Xu J. & Li Y. Discovering disease-genes by topological features in human protein-protein interaction network. Bioinformatics 22, 2800–5 (2006). 16954137 10.1093/bioinformatics/btl467

[b13] Cai J. J., Borenstein E. & Petrov D. A. Broker genes in human disease. Genome Biol. Evol. 2, 815–25 (2010). 20937604 10.1093/gbe/evq064 PMC2988523

[b14] Goh K.-I. . The human disease network. Proc. Natl. Acad. Sci. USA 104, 8685–90 (2007). 17502601 10.1073/pnas.0701361104 PMC1885563

[b15] Furlong L. I. Human diseases through the lens of network biology. Trends Genet. **null** (2012). 10.1016/j.tig.2012.11.004 23219555

[b16] Piñero J. . DisGeNET: a discovery platform for the dynamical exploration of human diseases and their genes. Database 2015, bav028–bav028 (2015). 25877637 10.1093/database/bav028 PMC4397996

[b17] Garcia-Alonso L. . The role of the interactome in the maintenance of deleterious variability in human populations. Mol. Syst. Biol. 10, 752 (2014). 25261458 10.15252/msb.20145222 PMC4299661

[b18] Schaefer M. H. . HIPPIE: Integrating protein interaction networks with experiment based quality scores. Plos One 7, e31826 (2012). 22348130 10.1371/journal.pone.0031826 PMC3279424

[b19] Rual J.-F. . Towards a proteome-scale map of the human protein-protein interaction network. Nature 437, 1173–8 (2005). 16189514 10.1038/nature04209

[b20] Rosvall M. & Bergstrom C. T. Maps of random walks on complex networks reveal community structure. Proc. Natl. Acad. Sci. USA 105, 1118–23 (2008). 18216267 10.1073/pnas.0706851105 PMC2234100

[b21] Lancichinetti A. & Fortunato S. Community detection algorithms: A comparative analysis. Phys. Rev. E 80, 056117 (2009). 10.1103/PhysRevE.80.056117 20365053

[b22] Berenstein A. J., Piñero J., Furlong L. I. & Chernomoretz A. Mining the modular structure of protein interaction networks. Plos One 10, e0122477 (2015). 25856434 10.1371/journal.pone.0122477 PMC4391834

[b23] Liu W., Pellegrini M. & Wang X. Detecting communities based on network topology. Sci. Rep. 4, 5739 (2014). 25033828 10.1038/srep05739 PMC4102921

[b24] Guimerà R. & Amaral L. A. N. Cartography of complex networks: modules and universal roles. J. Stat. Mech. Online 2005, nihpa35573 (2005). 10.1088/1742-5468/2005/02/P02001 PMC2151742 18159217

[b25] Guimerà R. & Amaral L. A. N. Functional cartography of complex metabolic networks. Nature 433, 895–900 (2005). 15729348 10.1038/nature03288 PMC2175124

[b26] Thomas P. D. PANTHER: a browsable database of gene products organized by biological function, using curated protein family and subfamily classification. Nucleic Acids Res. 31, 334–341 (2003). 12520017 10.1093/nar/gkg115 PMC165562

[b27] Zhu X., Need A. C., Petrovski S. & Goldstein D. B. One gene, many neuropsychiatric disorders: lessons from Mendelian diseases. Nat. Neurosci. 17, 773–81 (2014). 24866043 10.1038/nn.3713

[b28] Kircher M. . A general framework for estimating the relative pathogenicity of human genetic variants. Nat. Genet. 46, 310–5 (2014). 24487276 10.1038/ng.2892 PMC3992975

[b29] Fernández-Medarde A. & Santos E. Ras in cancer and developmental diseases. Genes Cancer 2, 344–58 (2011). 21779504 10.1177/1947601911411084 PMC3128640

[b30] Menche J. . Disease networks. Uncovering disease-disease relationships through the incomplete interactome. Science 347, 1257601 (2015). 25700523 10.1126/science.1257601 PMC4435741

[b31] Hanahan D. & Weinberg R. A. The Hallmarks of Cancer. Cell 100, 57–70 (2000). 10647931 10.1016/s0092-8674(00)81683-9

[b32] Hanahan D. & Weinberg R. A. Hallmarks of cancer: the next generation. Cell 144, 646–74 (2011). 21376230 10.1016/j.cell.2011.02.013

[b33] Vogelstein B. . Cancer genome landscapes. Science 339, 1546–58 (2013). 23539594 10.1126/science.1235122 PMC3749880

[b34] Hao D. . Systematic large-scale study of the inheritance mode of Mendelian disorders provides new insight into human diseasome. Eur. J. Hum. Genet. doi: 10.1038/ejhg.2013.309 (2014). PMC4200425 24448549

[b35] Hao D. . Network-based analysis of genotype-phenotype correlations between different inheritance modes. Bioinformatics 30, 3223–31 (2014). 25078399 10.1093/bioinformatics/btu482

[b36] Veitia R. A. Exploring the etiology of haploinsufficiency. Bioessays 24, 175–84 (2002). 11835282 10.1002/bies.10023

[b37] Wilkie A. O. The molecular basis of genetic dominance. J. Med. Genet. 31, 89–98 (1994). 8182727 10.1136/jmg.31.2.89 PMC1049666

[b38] Petrovski S., Wang Q., Heinzen E. L., Allen A. S. & Goldstein D. B. Genic intolerance to functional variation and the interpretation of personal genomes. PLoS Genet. 9, e1003709 (2013). 23990802 10.1371/journal.pgen.1003709 PMC3749936

[b39] Shyr C. . FLAGS, frequently mutated genes in public exomes. BMC Med. Genomics 7, 64 (2014). 25466818 10.1186/s12920-014-0064-y PMC4267152

[b40] Garcia-Garcia J., Guney E., Aragues R., Planas-Iglesias J. & Oliva B. Biana: a software framework for compiling biological interactions and analyzing networks. BMC Bioinformatics 11, 56 (2010). 20105306 10.1186/1471-2105-11-56 PMC3098100

[b41] Stark C. . BioGRID: a general repository for interaction datasets. Nucleic Acids Res. 34, D535–9 (2006). 16381927 10.1093/nar/gkj109 PMC1347471

[b42] Orchard S. . The MIntAct project–IntAct as a common curation platform for 11 molecular interaction databases. Nucleic Acids Res. 42, D358–63 (2014). 24234451 10.1093/nar/gkt1115 PMC3965093

[b43] Razick S., Magklaras G. & Donaldson I. M. iRefIndex: a consolidated protein interaction database with provenance. BMC Bioinformatics 9, 405 (2008). 18823568 10.1186/1471-2105-9-405 PMC2573892

[b44] Rolland T. . A Proteome-Scale Map of the Human Interactome Network. Cell 159, 1212–1226 (2014). 25416956 10.1016/j.cell.2014.10.050 PMC4266588

[b45] Janjić V. & Pržulj N. Biological function through network topology: a survey of the human diseasome. Brief. Funct. Genomics 11, 522–32 (2012). 22962330 10.1093/bfgp/els037 PMC7109924

[b46] Wodak S. J., Vlasblom J., Turinsky A. L. & Pu S. Protein-protein interaction networks: the puzzling riches. Curr. Opin. Struct. Biol. 23, 941–53 (2013). 24007795 10.1016/j.sbi.2013.08.002

[b47] Lopes T. J. S. . Tissue-specific subnetworks and characteristics of publicly available human protein interaction databases. Bioinformatics 27, 2414–21 (2011). 21798963 10.1093/bioinformatics/btr414

[b48] Jensen L. J. & Bork P. Biochemistry. Not comparable, but complementary. Science 322, 56–7 (2008). 18832636 10.1126/science.1164801

[b49] Stelzl U. . A human protein-protein interaction network: a resource for annotating the proteome. Cell 122, 957–68 (2005). 16169070 10.1016/j.cell.2005.08.029

[b50] Ewing R. M. . Large-scale mapping of human protein-protein interactions by mass spectrometry. Mol. Syst. Biol. 3, 89 (2007). 17353931 10.1038/msb4100134 PMC1847948

[b51] Venkatesan K. . An empirical framework for binary interactome mapping. Nat. Methods 6, 83–90 (2009). 19060904 10.1038/nmeth.1280 PMC2872561

[b52] Wang J. . Toward an understanding of the protein interaction network of the human liver. Mol. Syst. Biol. 7, 536 (2011). 21988832 10.1038/msb.2011.67 PMC3261708

[b53] Kristensen A. R., Gsponer J. & Foster L. J. A high-throughput approach for measuring temporal changes in the interactome. Nat. Methods 9, 907–9 (2012). 22863883 10.1038/nmeth.2131 PMC3954081

[b54] Havugimana P. C. . A census of human soluble protein complexes. Cell 150, 1068–81 (2012). 22939629 10.1016/j.cell.2012.08.011 PMC3477804

[b55] Wagner S. A. . A proteome-wide, quantitative survey of *in vivo* ubiquitylation sites reveals widespread regulatory roles. Mol. Cell. Proteomics 10, M111.013284 (2011). 10.1074/mcp.M111.013284 PMC3205876 21890473

[b56] Stes E. . A COFRADIC protocol to study protein ubiquitination. J. Proteome Res. 13, 3107–13 (2014). 24816145 10.1021/pr4012443

[b57] Povlsen L. K. . Systems-wide analysis of ubiquitylation dynamics reveals a key role for PAF15 ubiquitylation in DNA-damage bypass. Nat. Cell Biol. 14, 1089–98 (2012). 23000965 10.1038/ncb2579

[b58] Bandyopadhyay S. . A human MAP kinase interactome. Nat. Methods 7, 801–5 (2010). 20936779 10.1038/nmeth.1506 PMC2967489

[b59] Vinayagam A. . A directed protein interaction network for investigating intracellular signal transduction. Sci. Signal. 4, rs8 (2011). 21900206 10.1126/scisignal.2001699

[b60] Futreal P. A. . A census of human cancer genes. Nat. Rev. Cancer 4, 177–83 (2004). 14993899 10.1038/nrc1299 PMC2665285

[b61] Rubio-Perez C. . *In Silico* Prescription of Anticancer Drugs to Cohorts of 28 Tumor Types Reveals Targeting Opportunities. Cancer Cell 27, 382–396 (2015). 25759023 10.1016/j.ccell.2015.02.007

[b62] Amberger J., Bocchini C. A., Scott A. F. & Hamosh A. McKusick’s Online Mendelian Inheritance in Man (OMIM). Nucleic Acids Res 37, D793–6 (2009). 18842627 10.1093/nar/gkn665 PMC2686440

[b63] Singh P. P., Affeldt S., Malaguti G. & Isambert H. Human dominant disease genes are enriched in paralogs originating from whole genome duplication. PLoS Comput. Biol. 10, e1003754 (2014). 25080083 10.1371/journal.pcbi.1003754 PMC4117431

[b64] Blekhman R. . Natural selection on genes that underlie human disease susceptibility. Curr. Biol. 18, 883–9 (2008). 18571414 10.1016/j.cub.2008.04.074 PMC2474766

[b65] The UniProt Consortium. Activities at the Universal Protein Resource (UniProt). Nucleic Acids Res. 42, D191–8 (2014). 24253303 10.1093/nar/gkt1140 PMC3965022

[b66] Landrum M. J. . ClinVar: public archive of relationships among sequence variation and human phenotype. Nucleic Acids Res. 42, D980–5 (2014). 24234437 10.1093/nar/gkt1113 PMC3965032

[b67] Mi H. & Thomas P. PANTHER pathway: an ontology-based pathway database coupled with data analysis tools. Methods Mol. Biol. Clift. Nj 563, 123–140 (2009). 10.1007/978-1-60761-175-2_7 PMC6608593 19597783

[b68] R Core Team. R: A Language and Environment for Statistical Computing. R Foundation for Statistical Computing, Vienna, Austria. URL http://www.R-project.org/.

[b69] Csardi G. & Nepusz T. The igraph software package for complex network research. *InterJournal Complex Systems* 1695 URL http://igraph.org/ (2006).

[b70] Falcon S. & Gentleman R. Using GOstats to test gene lists for GO term association. Bioinformatics 23, 257–8 (2007). 17098774 10.1093/bioinformatics/btl567

