# Supplementary material for: Uncovering disease mechanisms through network biology in the era of Next Generation Sequencing
Source: Sci Rep. 2016 Apr 15;6:24570. doi: 10.1038/srep24570 (PMC4832203; doi:10.1038/srep24570)
Supplement: Supplementary Information [file srep24570-s1.pdf]

Supplementary materials for

Uncovering disease mechanisms through network biology in the era of Next Generation Sequencing

Janet Piñero, Ariel Berenstein, Abel Gonzalez-Perez, Ariel Chernomoretz, Laura I. Furlong

This document contains: Supplementary Figures S1–S10 and supplementary Tables S1–S5.

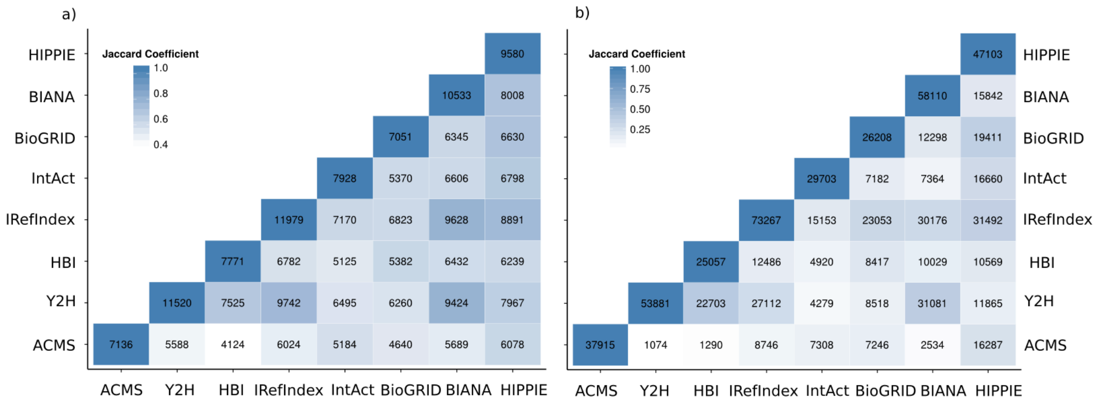

Supplementary Figure S1: Overlaps between PINs in terms of proteins (Panel a) and interactions (Panel b), measured with the Jaccard coefficient.

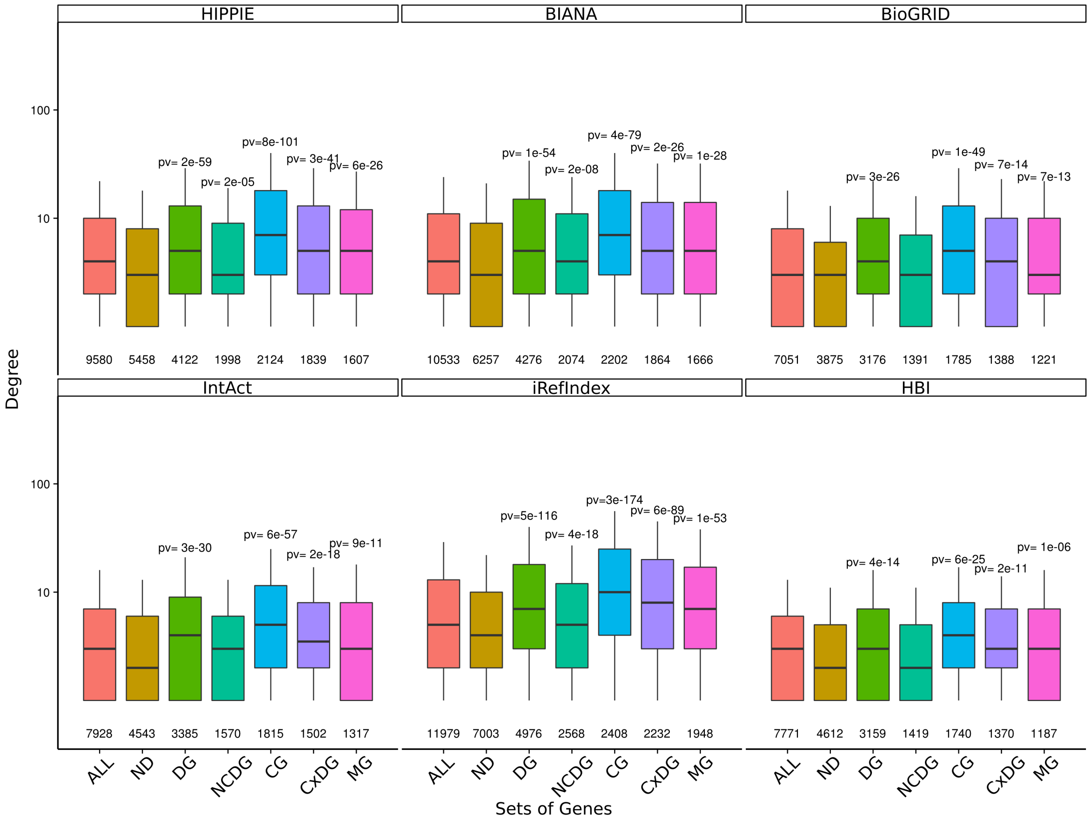

Supplementary Figure S2: Degree distribution of the different sets of disease genes across all the protein interaction networks.

ALL: all genes, ND: non-disease genes, DG: all disease genes, NCDG: non-cancer disease genes, CG: cancer genes, CxDG: complex disease genes, MG: Mendelian disease genes. We show the p-values of Man-Whitney test resulting from comparisons of the different groups of disease genes with non-disease genes, corrected by multiple testing according to Benjamini & Hochberg method.

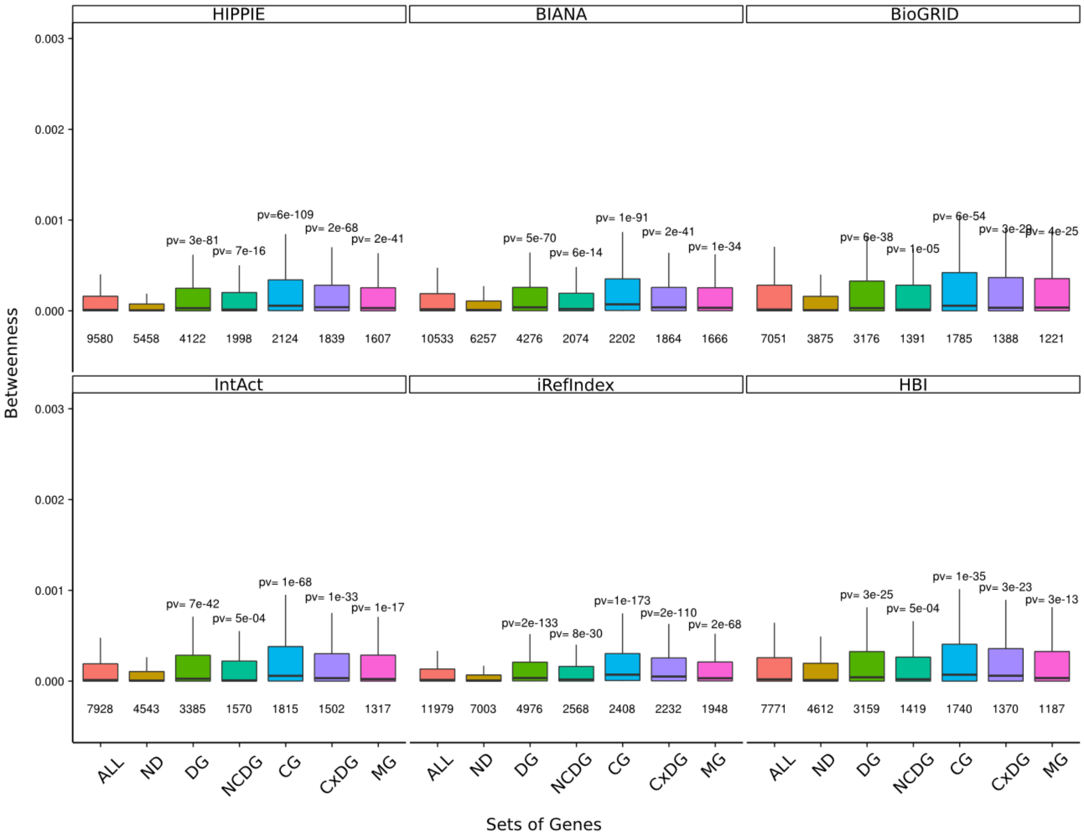

Supplementary Figure S3: Betweenness distribution of the different sets of disease genes across all the protein interaction networks.

ALL: all genes, ND: non-disease genes, DG: all disease genes, NCDG: non-cancer disease genes, CG: cancer genes, CxDG: complex disease genes, MG: Mendelian disease genes. We show the p-values of Man-Whitney test resulting from comparisons of the different groups of disease genes with non-disease genes, corrected by multiple testing according to Benjamini & Hochberg method.

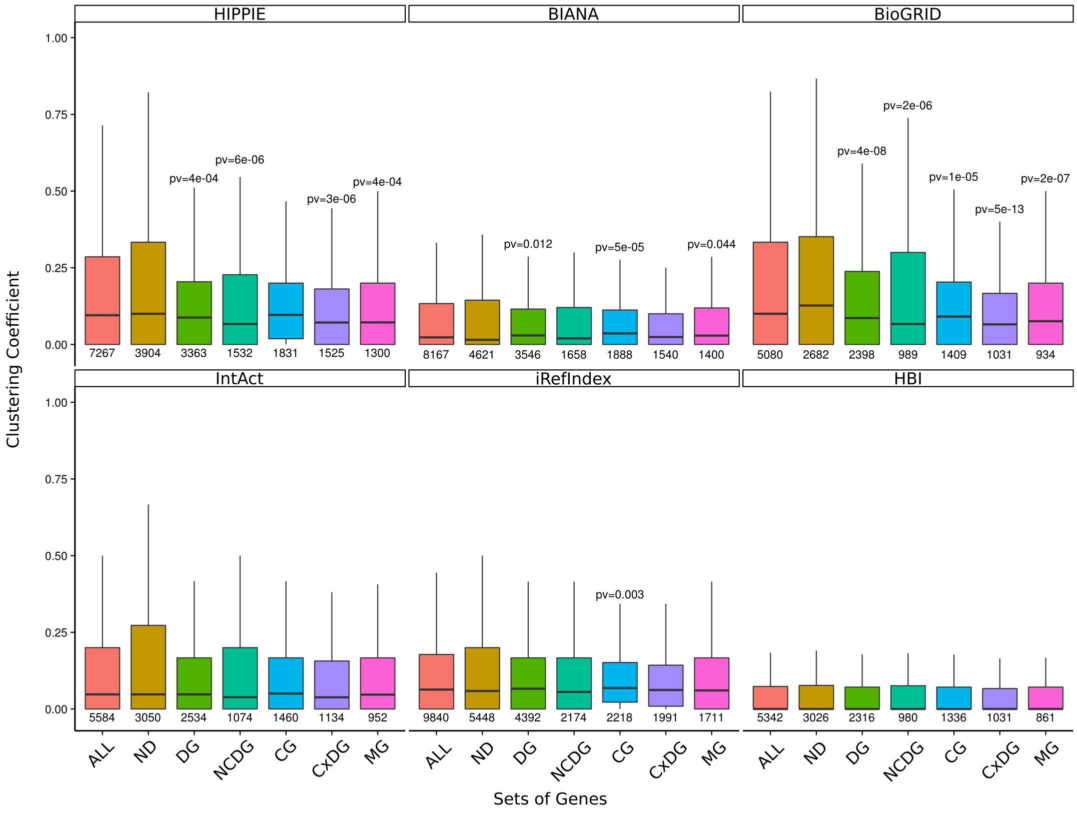

Supplementary Figure S4: Clustering coefficient (CC) distribution of the different sets of disease genes across all the protein interaction networks.

ALL: all genes, ND: non-disease genes, DG: all disease genes, NCDG: non-cancer disease genes, CG: cancer genes, CxDG: complex disease genes, MG: Mendelian disease genes. We show the p-values of Man-Whitney test resulting from comparisons of the different groups of disease genes with non-disease genes, corrected by multiple testing according to Benjamini & Hochberg method.

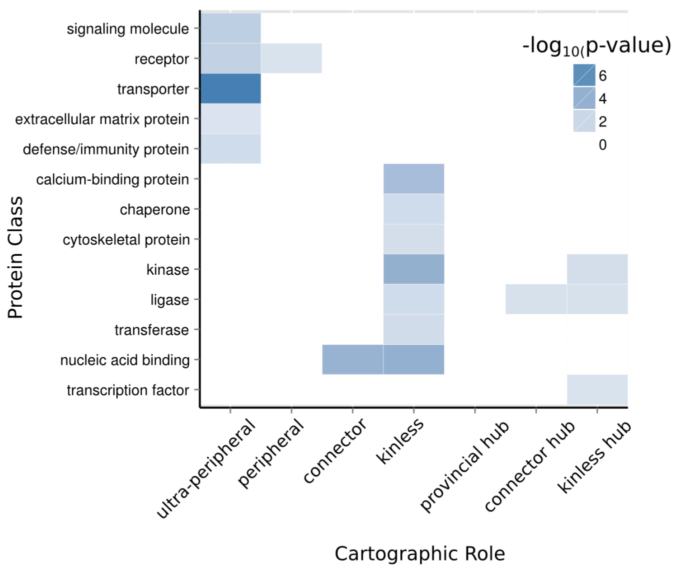

Supplementary Figure S5: Overrepresentation of Panther protein classes in each cartographic role in the HIPPIE protein interaction network.

The color scale is proportional to the p-value of the exact Fisher test, corrected for multiple testing by Benjamini & Hochberg.

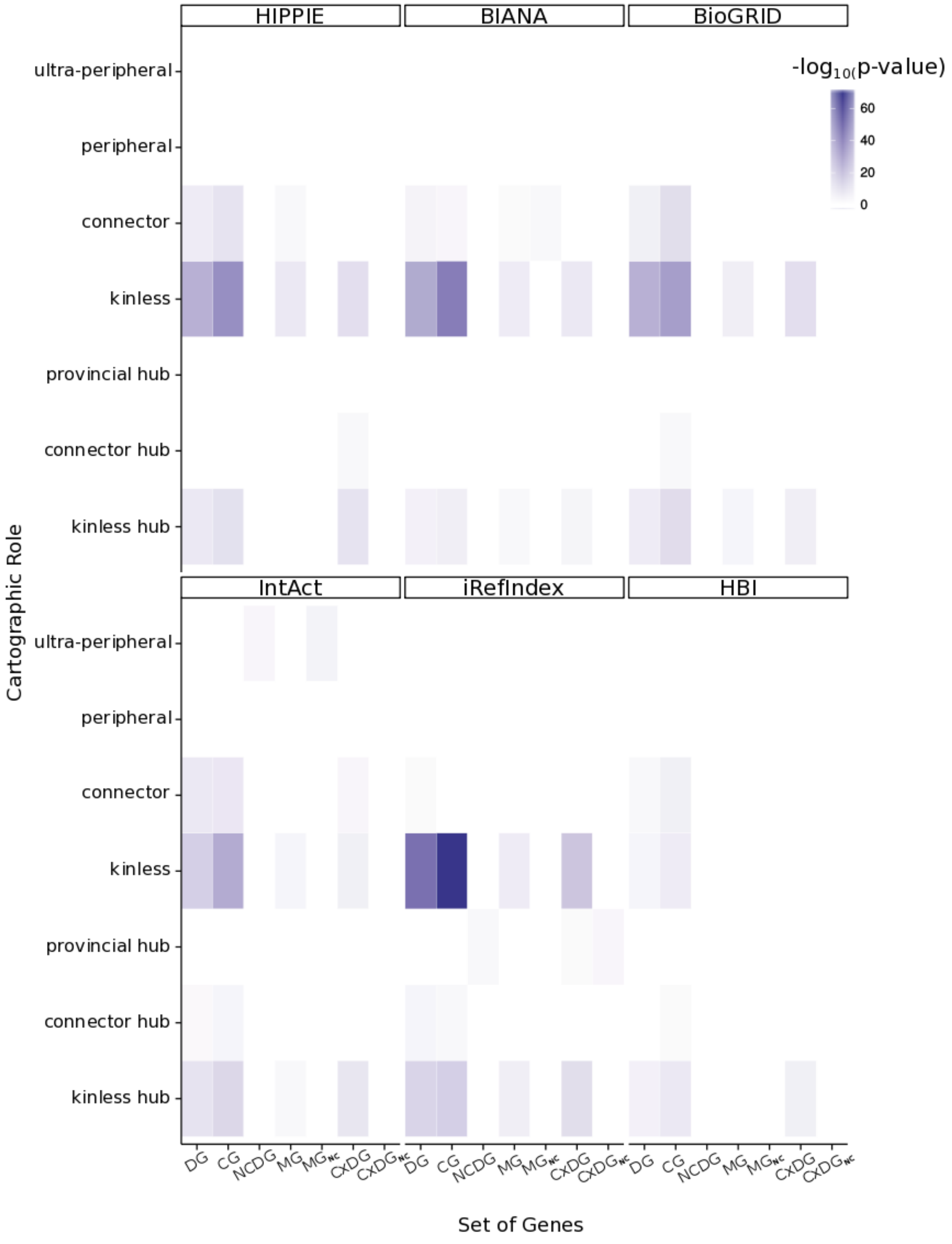

Supplementary Figure S6: Overrepresentation of the different sets of disease genes in each cartographic role across all the protein interaction networks.

DG: all disease genes, NCDG: non-cancer disease genes, CG: cancer genes, CxDG: complex disease genes, $CxDG_{NC}$: CxDG without cancer genes, MG: Mendelian disease

genes, MG_{NC}: MG without cancer genes. The color is proportional to logarithm of p-value of the exact Fisher test, corrected for multiple testing by Benjamini & Hochberg.

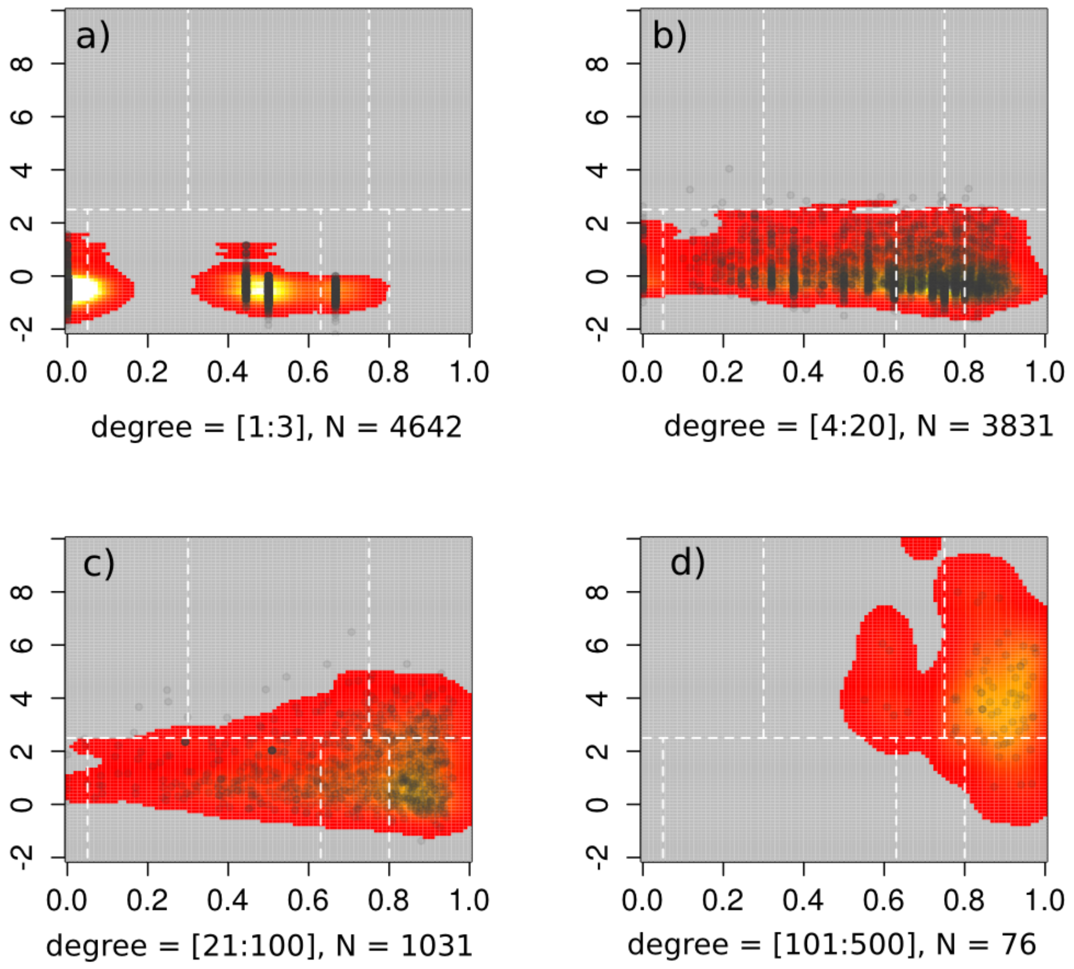

Figure S7: The color-coded kernel density estimation of the distribution of HIPPIE genes divided in four degree intervals over the z-P plane.

The distribution of PIN genes over the z-P plane for four degree intervals [1,3], [4,20], [21,100] and [101,500] is shown in panels (a), (b), (c) and (d) respectively. A color-coded kernel density estimation was calculated for the 4642, 3831, 1031 and 76 genes included in each panel. Dashed lines in the figures delineate regions corresponding to the seven cartographic roles.

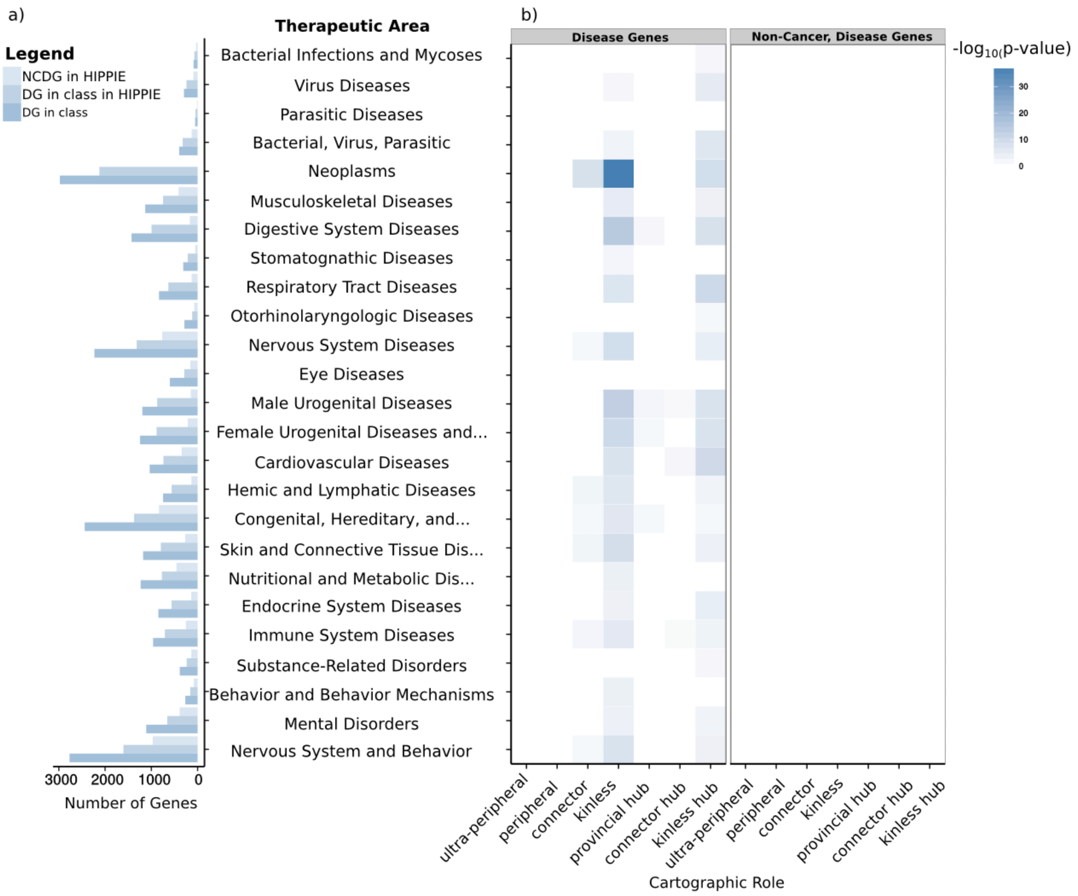

Supplementary Figure S8: Overrepresentation of the different sets of genes belonging to the MeSH disease classifications in each cartographic role in the HIPPIE protein interaction network.

a) Distribution of disease genes in each MeSH category. DG in class: Total number of genes in MeSH category, DG in class in HIPPIE: DG in class that map to HIPPIE, NCDG: non-cancer disease genes in class that map to HIPPIE.

b) Overrepresentation of the different groups of diseases genes in each cartographic role. The color is proportional to logarithm of the p-value of the exact Fisher test, corrected for multiple testing by Benjamini & Hochberg.

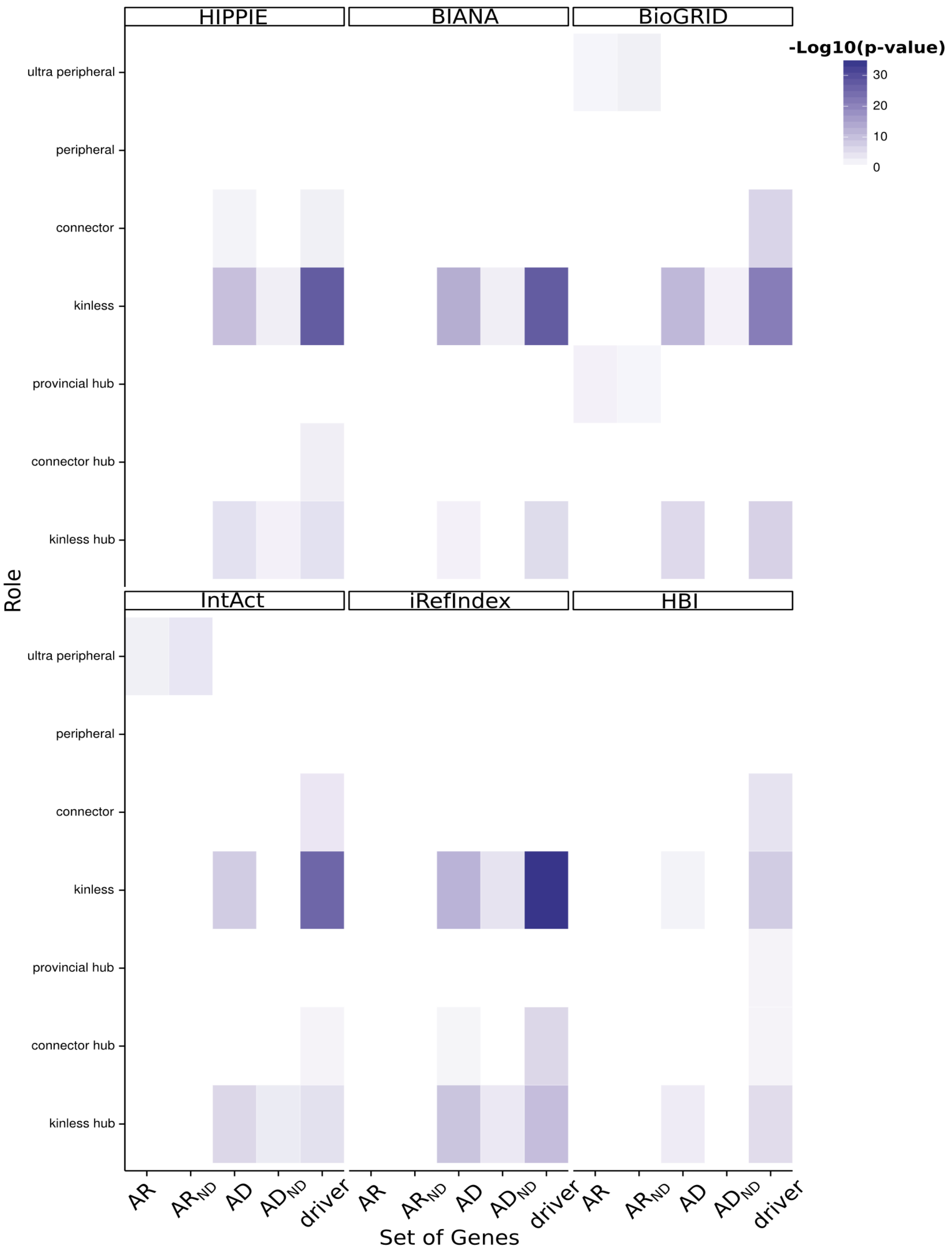

Supplementary Figure S9: Overrepresentation of the different sets of disease genes in each cartographic role across the all the protein interaction networks.

AD: Autosomal Dominant, AD_{ND}: AD genes without driver genes, AR: Autosomal Recessive, AR_{ND}: AR genes without driver genes, driver: cancer driver genes. The color is proportional to logarithm of p-value of the exact Fisher test, corrected for multiple testing by Benjamini & Hochberg.

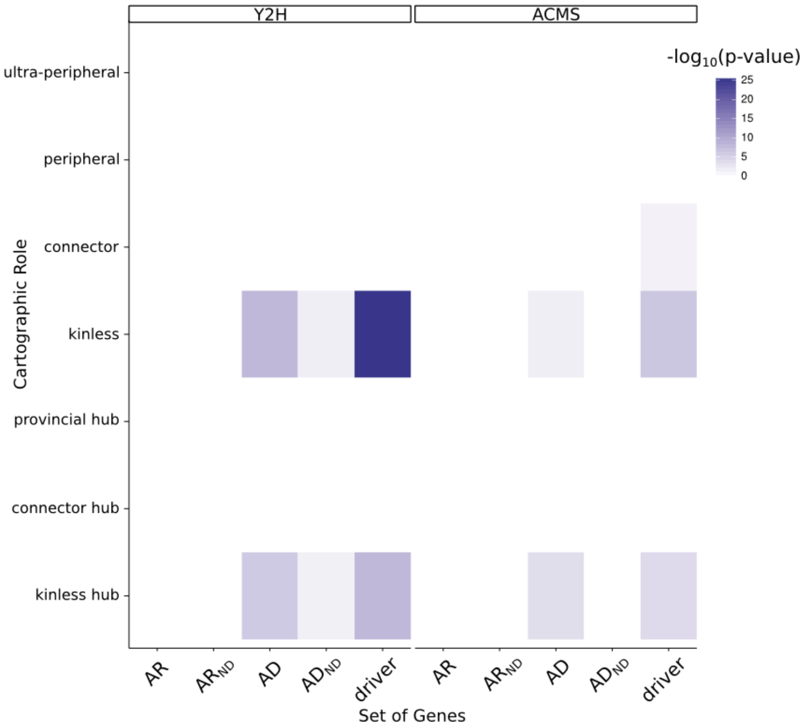

Supplementary Figure S10: Overrepresentation of the different sets of disease genes in each cartographic role in the Y2H and ACMS protein interaction networks

AD: Autosomal Dominant, AD_{ND}: AD genes without driver genes, AR: Autosomal Recessive, AR_{ND}: AR genes without driver genes, driver: cancer driver genes. The color is proportional to logarithm of p-value of the exact Fisher test, corrected for multiple testing by Benjamini & Hochberg.

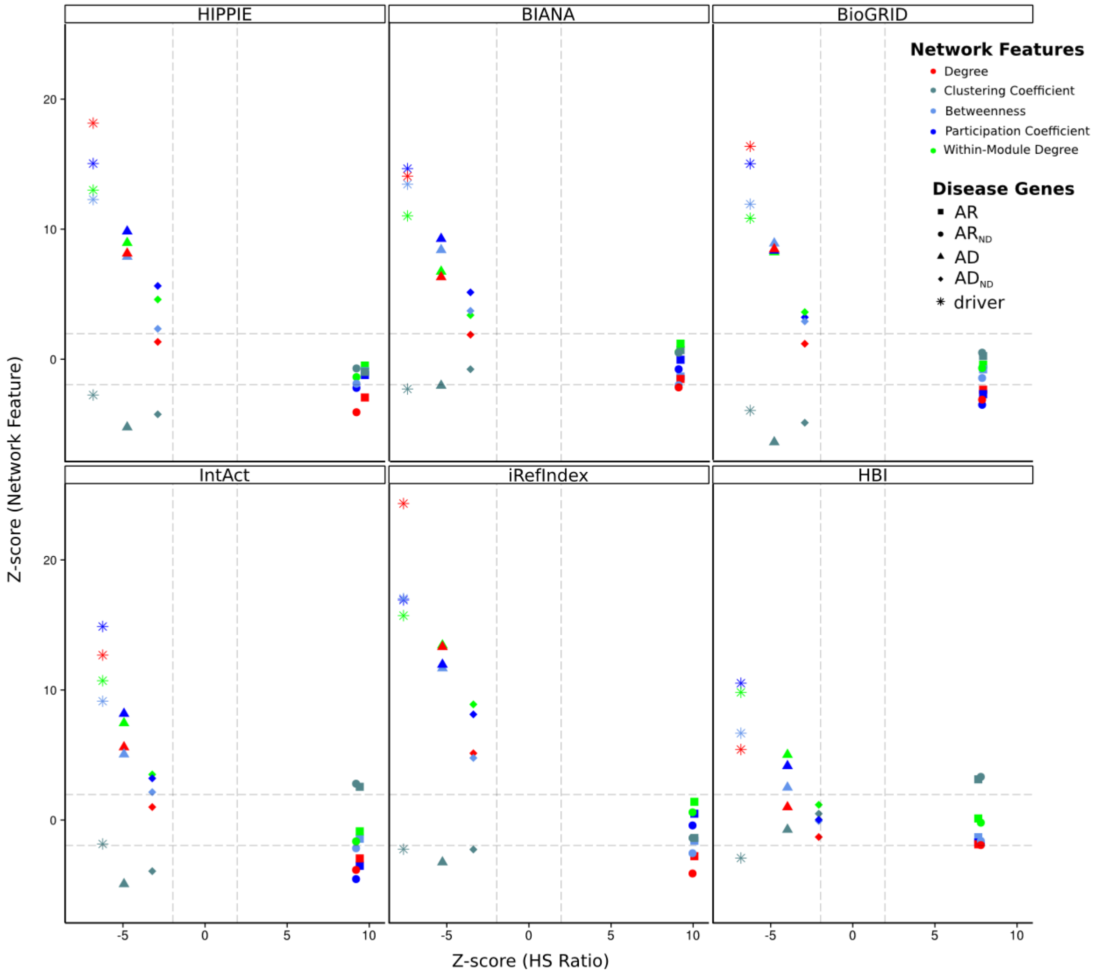

Supplementary Figure S11: Relationship between network features (degree, betweenness, clustering coefficient, participation coefficient, and within-module degree) and the HS Ratio for the disease gene sets across all protein interaction networks

We plot the z-scores resulting from 10,000 randomizations. AD: Autosomal Dominant, AD_{ND}: AD genes without driver genes, AR: Autosomal Recessive, AR_{ND}: AR genes without driver genes, driver: cancer driver genes.

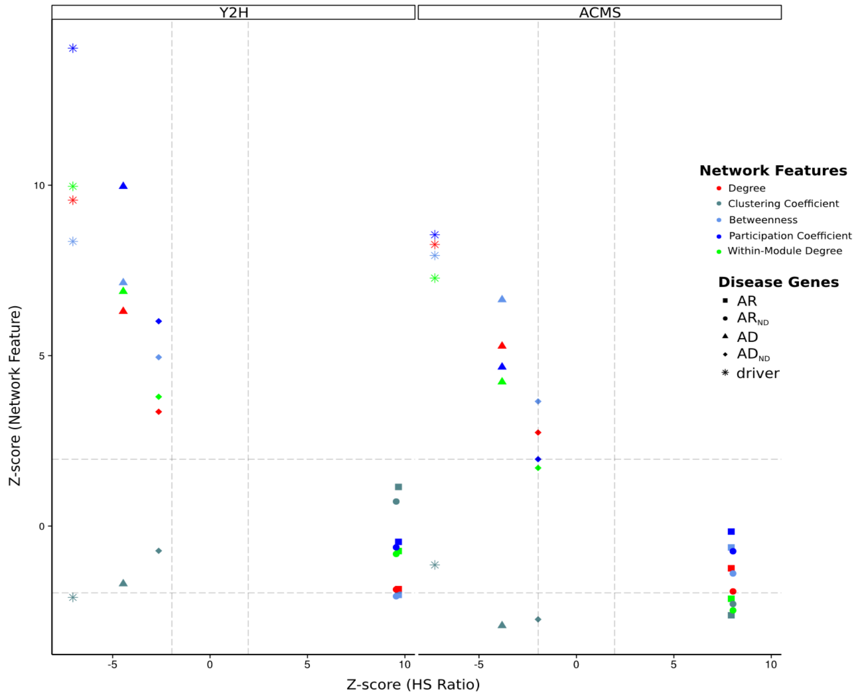

Supplementary Figure S12: Relationship between network features (degree, betweenness, clustering coefficient, participation coefficient, and within-module degree) and the HS Ratio for the disease gene sets in the Y2H and ACMS protein interaction networks.

We plot the z-scores resulting from 10,000 randomizations. AD: Autosomal Dominant, AD_{ND}: AD genes without driver genes, AR: Autosomal Recessive, AR_{ND}: AR genes without driver genes, driver: cancer driver genes.

Supplementary Table S1: Detailed description of the protein interaction networks employed in this study

	HIPPIE	BioGRID	IntAct	BIANA	HBI	iRefIndex	Y2H	ACMS
**Nodes**	9580	7051	7928	10533	7771	11979	11520	7136
**Interactions**	47103	26208	29703	58110	25057	73267	53881	37915
**Degree**	9,8	7,4	7,5	11,0	6,4	12,2	9,4	10,6
**Betweenness**	0,0003	0,0005	0,0004	0,0003	0,0004	0,0002	0,0003	0,0004
**CC**	0,198	0,221	0,166	0,122	0,091	0,141	0,074	0,199
**Shortest Path**	4,0	4,3	4,1	4,0	4,4	3,8	4,0	3,6
**Diameter**	12	12	13	11	13	14	10	10
**Unbiased**	4,3	5,5	2,2	5,0	61,4	4,2	28,5	0,5
**Biased High-throughput**	65,9	50,3	72,9	66,2	23,6	63,1	52,3	78,6
**Biased Low-throughput**	61,4	80,0	43,5	33,7	34,0	57,7	23,1	30,4
**Biased Low-throughput only**	29,8	44,2	24,9	20,6	14,9	32,7	13,5	20,9

For each protein interaction network, we show the number of nodes, interactions, and the average degree, betweenness, CC, shortest path, and diameter. In addition, we show the percentage of unbiased interactions (i.e. interactions that do not interrogate specific portions of the interactome, reported by references 19, 44, 49, 50, 51, 52, 53, and 54) covered by each network. The rest of the interactions were considered as "biased". From this set, we computed the percentage of interactions reported by high-throughput studies (papers reporting 100 or more interactions), and low-throughput studies (papers reporting less than 100 interactions). "low-throughput only" refers to the percentage of the interactions in the network that are supported only by low-throughput studies.

Supplementary Table S2: Percent of genes in each disease classification that is included in the protein interaction network.

Gene Set	Abb.	size	HIPPIE	BioGRID	IntAct	BIANA	HBI	iRefIndex	Y2H	ACMS
**Disease Genes**	DG	7412	55,6	42,8	45,7	57,7	42,6	67,1	58,7	37,9
**Cancer related Genes**	CG	2977	71,3	60,0	61,0	74,0	58,4	80,9	74,9	51,9
**Non-cancer disease genes**	NCDG	4435	45,1	31,4	35,4	46,8	32,0	57,9	47,9	28,6
**Mendelian Genes**	MG	3114	51,6	39,2	42,3	53,5	38,1	62,6	53,5	35,4
**Complex Disease Genes**	CxDG	2863	64,2	48,5	52,5	65,1	47,9	78,0	64,9	38,8
**Autosomal Recessive Disease Genes**	AR	1153	59,3	41,5	47,4	65,6	39,0	78,5	63,2	41,4
**AR non-drivers**	AR_{ND}	1106	58,0	39,4	45,9	64,6	37,7	77,8	62,2	40,4
**Autosomal Dominant Disease Genes**	AD	954	78,6	63,2	63,5	77,1	64,4	89,2	78,8	51,4
**AD non-drivers**	AD_{ND}	787	74,8	57,1	59,0	74,0	59,1	87,3	75,5	46,8
**Driver Genes**	drivers	781	88,5	81,6	78,7	86,8	76,3	92,7	89,5	72,5

Supplementary Table S3: Percent of genes in the seven cartographic roles in each protein interaction network.

Cartographic Role	HIPPIE	BioGRID	IntAct	BIANA	HBI	iRefIndex	ACMS	Y2H
**ultra-peripheral**	34,9	42,2	42,3	29,9	43,0	25,4	38,1	30,9
**peripheral**	27,1	27,0	26,1	25,6	27,4	25,5	26,1	25,0
**connector**	22,9	18,9	20,6	25,1	20,7	26,5	23,7	26,1
**kinless**	12,3	8,7	7,9	17,0	6,2	19,9	9,1	15,0
**provincial hub**	0,1	0,2	0,1	0,1	0,1	0,1	0,0	0,0
**connector hub**	0,7	1,1	1,2	0,5	1,2	0,7	1,4	0,9
**kinless hub**	2,0	1,8	1,8	1,8	1,4	1,9	1,7	2,1

Supplementary Table S4: Enrichment analysis of each cartographic role in HIPPIE for the Gene Ontology (GO) molecular function.

GOMFID	p-value	OR	Term	Cartographic role
GO:0000989	5E-18	2,813	transcription factor binding transcription factor activity	connector
GO:0003714	2E-10	2,950	transcription corepressor activity	connector
GO:0016874	6E-10	2,042	ligase activity	connector
GO:0016772	1E-09	1,720	transferase activity, transferring phosphorus-containing groups	connector
GO:0016881	7E-09	2,296	acid-amino acid ligase activity	connector
GO:0004842	8E-09	2,398	ubiquitin-protein ligase activity	connector
GO:0003713	1E-08	2,495	transcription coactivator activity	connector
GO:0004386	5E-06	2,485	helicase activity	connector
GO:0016817	7E-06	1,543	hydrolase activity, acting on acid anhydrides	connector
GO:0016462	7E-06	1,545	pyrophosphatase activity	connector
GO:0004672	7E-05	2,220	protein kinase activity	connector
GO:0008656	0,0002	6,534	cysteine-type endopeptidase activator activity involved in apoptotic process	connector
GO:0016876	0,0003	3,505	ligase activity, forming aminoacyl-tRNA and related compounds	connector
GO:0003924	0,0005	1,822	GTPase activity	connector
GO:0008026	0,0006	2,554	ATP-dependent helicase activity	connector
GO:0004674	0,0006	1,599	protein serine/threonine kinase activity	connector
GO:0034062	0,0007	3,270	RNA polymerase activity	connector
GO:0005200	0,0012	2,215	structural constituent of cytoskeleton	connector
GO:0003735	0,0013	1,907	structural constituent of ribosome	connector
GO:0016504	0,0013	3,410	peptidase activator activity	connector
GO:0005057	0,0015	2,705	receptor signaling protein activity	connector
GO:0004715	0,0016	2,953	non-membrane spanning protein tyrosine kinase activity	connector
GO:0005096	0,0019	1,643	GTPase activator activity	connector
GO:0003705	0,0022	2,182	RNA polymerase II distal enhancer sequence-specific DNA binding transcription fa...	connector
GO:0004708	0,0042	4,569	MAP kinase kinase activity	connector
GO:0016887	0,0044	1,474	ATPase activity	connector
GO:0005092	0,0058	6,529	GDP-dissociation inhibitor activity	connector
GO:0015271	0,0059	6,523	outward rectifier potassium channel activity	connector
GO:0016278	0,0069	2,498	lysine N-methyltransferase activity	connector
GO:0001618	0,0083	3,093	virus receptor activity	connector
GO:0030159	0,0094	4,358	receptor signaling complex scaffold activity	connector
GO:0001056	0,0095	4,349	RNA polymerase III activity	connector
GO:0000146	0,0117	3,514	microfilament motor activity	connector
GO:0008307	0,0126	2,375	structural constituent of muscle	connector
GO:0030374	0,0126	2,375	ligand-dependent nuclear receptor transcription coactivator activity	connector
GO:0042800	0,0147	4,659	histone methyltransferase activity (H3-K4 specific)	connector
GO:0004702	0,015	2,136	receptor signaling protein serine/threonine kinase activity	connector
GO:0004702	0,015	2,136	receptor signaling protein serine/threonine kinase activity	connector
GO:0016538	0,0156	3,262	cyclin-dependent protein serine/threonine kinase regulator activity	connector

GO:0001076	0,0158	1,749	RNA polymerase II transcription factor binding transcription factor activity	connector
GO:0004714	0,0177	2,082	transmembrane receptor protein tyrosine kinase activity	connector
GO:0004714	0,0177	2,082	transmembrane receptor protein tyrosine kinase activity	connector
GO:0004714	0,0177	2,082	transmembrane receptor protein tyrosine kinase activity	connector
GO:0005487	0,0213	4,076	nucleocytoplasmic transporter activity	connector
GO:0003707	0,0225	2,071	steroid hormone receptor activity	connector
GO:0003707	0,0225	2,071	steroid hormone receptor activity	connector
GO:0030695	0,025	2,197	GTPase regulator activity	connector
GO:0004003	0,0251	2,447	ATP-dependent DNA helicase activity	connector
GO:0004843	0,0272	1,945	ubiquitin-specific protease activity	connector
GO:0004707	0,0295	3,623	MAP kinase activity	connector
GO:0004707	0,0295	3,623	MAP kinase activity	connector
GO:0030675	0,0295	3,623	Rac GTPase activator activity	connector
GO:0032947	0,0295	2,359	protein complex scaffold	connector
GO:0018024	0,0314	2,490	histone-lysine N-methyltransferase activity	connector
GO:0004029	0,0339	4,346	aldehyde dehydrogenase (NAD) activity	connector
GO:0004385	0,0339	4,346	guanylate kinase activity	connector
GO:0008047	0,0353	1,575	enzyme activator activity	connector
GO:0001071	0,0358	1,175	nucleic acid binding transcription factor activity	connector
GO:0019207	0,0359	1,557	kinase regulator activity	connector
GO:0004722	0,0376	1,958	protein serine/threonine phosphatase activity	connector
GO:0043027	0,0409	2,795	cysteine-type endopeptidase inhibitor activity involved in apoptotic process	connector
GO:0008276	0,0449	1,749	protein methyltransferase activity	connector
GO:0045182	0,0458	2,269	translation regulator activity	connector
GO:0004529	0,0477	3,725	exodeoxyribonuclease activity	connector
GO:0016303	0,0477	3,725	1-phosphatidylinositol-3-kinase activity	connector
GO:0030898	0,0477	3,725	actin-dependent ATPase activity	connector
GO:0030291	0,0488	2,403	protein serine/threonine kinase inhibitor activity	connector
GO:0004843	0,0002	16,409	ubiquitin-specific protease activity	connector hub
GO:0004842	0,0063	4,596	ubiquitin-protein ligase activity	connector hub
GO:0008234	0,0064	5,807	cysteine-type peptidase activity	connector hub
GO:0016881	0,0107	4,018	acid-amino acid ligase activity	connector hub
GO:0004672	0,0144	2,928	protein kinase activity	connector hub
GO:0016772	0,0187	2,426	transferase activity, transferring phosphorus-containing groups	connector hub
GO:0042578	0,0204	3,387	phosphoric ester hydrolase activity	connector hub
GO:0005057	0,0216	5,332	receptor signaling protein activity	connector hub
GO:0016818	0,0232	2,463	hydrolase activity, acting on acid anhydrides, in phosphorus-containing anhydrides	connector hub
GO:0017111	0,0476	2,243	nucleoside-triphosphatase activity	connector hub
GO:0000988	2E-20	3,240	protein binding transcription factor activity	kinless
GO:0016773	5E-16	2,585	phosphotransferase activity, alcohol group as acceptor	kinless
GO:0016301	3E-15	2,465	kinase activity	kinless
GO:0005200	4E-12	5,849	structural constituent of cytoskeleton	kinless
GO:0004674	9E-12	3,260	protein serine/threonine kinase activity	kinless
GO:0004843	2E-11	7,638	ubiquitin-specific protease activity	kinless
GO:0003714	3E-10	3,716	transcription corepressor activity	kinless
GO:0004221	9E-10	10,576	ubiquitin thiolesterase activity	kinless
GO:0003713	1E-09	3,004	transcription coactivator activity	kinless

GO:0004693	2E-08	9,752	cyclin-dependent protein serine/threonine kinase activity	kinless
GO:0034979	5E-07	16,063	NAD-dependent protein deacetylase activity	kinless
GO:0016881	6E-07	2,398	acid-amino acid ligase activity	kinless
GO:0001077	2E-06	4,261	RNA polymerase II core promoter proximal region sequence-specific DNA binding tr...	kinless
GO:0000981	3E-06	2,777	sequence-specific DNA binding RNA polymerase II transcription factor activity	kinless
GO:0043539	3E-06	21,830	protein serine/threonine kinase activator activity	kinless
GO:0004407	5E-06	10,219	histone deacetylase activity	kinless
GO:0004697	7E-06	12,481	protein kinase C activity	kinless
GO:0032041	7E-06	17,462	NAD-dependent histone deacetylase activity (H3-K14 specific)	kinless
GO:0016462	1E-05	1,700	pyrophosphatase activity	kinless
GO:0004842	1E-05	2,320	ubiquitin-protein ligase activity	kinless
GO:0008094	1E-05	6,588	DNA-dependent ATPase activity	kinless
GO:0016817	1E-05	1,685	hydrolase activity, acting on acid anhydrides	kinless
GO:0046969	1E-05	14,551	NAD-dependent histone deacetylase activity (H3-K9 specific)	kinless
GO:0001078	2E-05	6,251	RNA polymerase II core promoter proximal region sequence-specific DNA binding tr...	kinless
GO:0016740	2E-05	1,452	transferase activity	kinless
GO:0016874	2E-05	1,911	ligase activity	kinless
GO:0019789	3E-05	18,695	SUMO ligase activity	kinless
GO:0046970	6E-05	14,955	NAD-dependent histone deacetylase activity (H4-K16 specific)	kinless
GO:0008565	6E-05	3,436	protein transporter activity	kinless
GO:0019213	1E-04	5,430	deacetylase activity	kinless
GO:0097372	0,0001	12,461	NAD-dependent histone deacetylase activity (H3-K18 specific)	kinless
GO:0004197	0,0001	3,477	cysteine-type endopeptidase activity	kinless
GO:0019887	0,0001	4,204	protein kinase regulator activity	kinless
GO:0001105	0,0002	6,653	RNA polymerase II transcription coactivator activity	kinless
GO:0004712	0,0003	7,298	protein serine/threonine/tyrosine kinase activity	kinless
GO:0004857	0,0003	1,894	enzyme inhibitor activity	kinless
GO:0016887	0,0003	1,833	ATPase activity	kinless
GO:0035173	0,0004	12,483	histone kinase activity	kinless
GO:0001076	0,0004	2,645	RNA polymerase II transcription factor binding transcription factor activity	kinless
GO:0019209	0,0005	3,816	kinase activator activity	kinless
GO:0008234	0,0005	2,754	cysteine-type peptidase activity	kinless
GO:0030234	0,0009	1,629	enzyme regulator activity	kinless
GO:0003724	0,001	4,751	RNA helicase activity	kinless
GO:0003924	0,0011	1,990	GTPase activity	kinless
GO:0042054	0,0013	3,350	histone methyltransferase activity	kinless
GO:0004860	0,0018	3,193	protein kinase inhibitor activity	kinless
GO:0003705	0,0021	2,569	RNA polymerase II distal enhancer sequence-specific DNA binding transcription fa...	kinless
GO:0005095	0,0026	6,916	GTPase inhibitor activity	kinless
GO:0043027	0,0027	5,338	cysteine-type endopeptidase inhibitor activity involved in apoptotic process	kinless
GO:0000983	0,0045	8,293	RNA polymerase II core promoter sequence-specific DNA binding transcription fact...	kinless
GO:0001106	0,0045	4,670	RNA polymerase II transcription corepressor activity	kinless

GO:0008353	0,005	5,657	RNA polymerase II carboxy-terminal domain kinase activity	kinless
GO:0003755	0,0052	3,438	peptidyl-prolyl cis-trans isomerase activity	kinless
GO:0032947	0,0052	2,901	protein complex scaffold	kinless
GO:0070035	0,0063	2,173	purine NTP-dependent helicase activity	kinless
GO:0004708	0,0066	5,186	MAP kinase kinase activity	kinless
GO:0005198	0,0082	1,502	structural molecule activity	kinless
GO:0004861	0,0094	6,219	cyclin-dependent protein serine/threonine kinase inhibitor activity	kinless
GO:0004003	0,0097	3,353	ATP-dependent DNA helicase activity	kinless
GO:0008242	0,011	4,444	omega peptidase activity	kinless
GO:0003678	0,0126	5,551	DNA helicase activity	kinless
GO:0003712	0,0127	2,853	transcription cofactor activity	kinless
GO:0008080	0,0137	2,234	N-acetyltransferase activity	kinless
GO:0008170	0,0141	2,325	N-methyltransferase activity	kinless
GO:0004702	0,0154	2,557	receptor signaling protein serine/threonine kinase activity	kinless
GO:0004702	0,0154	2,557	receptor signaling protein serine/threonine kinase activity	kinless
GO:0015459	0,0156	3,006	potassium channel regulator activity	kinless
GO:0004683	0,017	3,888	calmodulin-dependent protein kinase activity	kinless
GO:0003700	0,018	1,311	sequence-specific DNA binding transcription factor activity	kinless
GO:0003756	0,0272	4,145	protein disulfide isomerase activity	kinless
GO:0004709	0,0296	3,274	MAP kinase kinase kinase activity	kinless
GO:0004709	0,0296	3,274	MAP kinase kinase kinase activity	kinless
GO:0004722	0,0376	2,264	protein serine/threonine phosphatase activity	kinless
GO:0004812	0,0384	2,420	aminoacyl-tRNA ligase activity	kinless
GO:0016860	0,0384	2,420	intramolecular oxidoreductase activity	kinless
GO:0016875	0,0384	2,420	ligase activity, forming carbon-oxygen bonds	kinless
GO:0005001	0,0406	3,552	transmembrane receptor protein tyrosine phosphatase activity	kinless
GO:0005001	0,0406	3,552	transmembrane receptor protein tyrosine phosphatase activity	kinless
GO:0005001	0,0406	3,552	transmembrane receptor protein tyrosine phosphatase activity	kinless
GO:0015036	0,0429	2,574	disulfide oxidoreductase activity	kinless
GO:0046933	0,0435	4,660	proton-transporting ATP synthase activity, rotational mechanism	kinless
GO:0046933	0,0435	4,660	proton-transporting ATP synthase activity, rotational mechanism	kinless
GO:0005057	0,0484	2,135	receptor signaling protein activity	kinless
GO:0003713	4E-09	6,432	transcription coactivator activity	kinless hub
GO:0000989	8E-09	5,219	transcription factor binding transcription factor activity	kinless hub
GO:0004842	1E-08	6,417	ubiquitin-protein ligase activity	kinless hub
GO:0016881	7E-08	5,576	acid-amino acid ligase activity	kinless hub
GO:0005057	4E-07	8,111	receptor signaling protein activity	kinless hub
GO:0016773	2E-06	3,309	phosphotransferase activity, alcohol group as acceptor	kinless hub
GO:0016301	2E-06	3,204	kinase activity	kinless hub
GO:0003700	2E-06	2,832	sequence-specific DNA binding transcription factor activity	kinless hub
GO:0046970	6E-06	49,345	NAD-dependent histone deacetylase activity (H4-K16 specific)	kinless hub
GO:0032041	8E-06	43,174	NAD-dependent histone deacetylase activity	kinless hub

			(H3-K14 specific)	
GO:0097372	8E-06	43,174	NAD-dependent histone deacetylase activity (H3-K18 specific)	kinless hub
GO:0004674	1E-05	3,976	protein serine/threonine kinase activity	kinless hub
GO:0046969	1E-05	38,375	NAD-dependent histone deacetylase activity (H3-K9 specific)	kinless hub
GO:0004707	2E-05	34,535	MAP kinase activity	kinless hub
GO:0004707	2E-05	34,535	MAP kinase activity	kinless hub
GO:0016874	2E-05	3,481	ligase activity	kinless hub
GO:0034979	3E-05	28,775	NAD-dependent protein deacetylase activity	kinless hub
GO:0004407	8E-05	21,576	histone deacetylase activity	kinless hub
GO:0001077	0,0002	7,564	RNA polymerase II core promoter proximal region sequence-specific DNA binding tr...	kinless hub
GO:0001076	0,0003	5,991	RNA polymerase II transcription factor binding transcription factor activity	kinless hub
GO:0050321	0,0003	28,620	tau-protein kinase activity	kinless hub
GO:0019213	0,0006	11,894	deacetylase activity	kinless hub
GO:0004197	0,0013	6,759	cysteine-type endopeptidase activity	kinless hub
GO:0001106	0,0021	13,548	RNA polymerase II transcription corepressor activity	kinless hub
GO:0016740	0,0021	1,824	transferase activity	kinless hub
GO:0004709	0,0027	12,256	MAP kinase kinase kinase activity	kinless hub
GO:0004709	0,0027	12,256	MAP kinase kinase kinase activity	kinless hub
GO:0004402	0,0034	7,030	histone acetyltransferase activity	kinless hub
GO:0016248	0,0042	10,293	channel inhibitor activity	kinless hub
GO:0004693	0,0061	8,871	cyclin-dependent protein serine/threonine kinase activity	kinless hub
GO:0003714	0,0151	3,155	transcription corepressor activity	kinless hub
GO:0004715	0,0156	6,120	non-membrane spanning protein tyrosine kinase activity	kinless hub
GO:0003678	0,0165	5,977	DNA helicase activity	kinless hub
GO:0098531	0,0196	5,586	direct ligand regulated sequence-specific DNA binding transcription factor activ...	kinless hub
GO:0016407	0,0228	3,904	acetyltransferase activity	kinless hub
GO:0004722	0,0229	5,243	protein serine/threonine phosphatase activity	kinless hub
GO:0016410	0,0245	3,817	N-acyltransferase activity	kinless hub
GO:0004843	0,0345	4,427	ubiquitin-specific protease activity	kinless hub
GO:0016810	0,0473	3,063	hydrolase activity, acting on carbon-nitrogen (but not peptide) bonds	kinless hub
GO:0019199	4E-07	13,483	transmembrane receptor protein kinase activity	peripheral
GO:0019199	4E-07	13,483	transmembrane receptor protein kinase activity	peripheral
GO:0019199	4E-07	13,483	transmembrane receptor protein kinase activity	peripheral
GO:0004298	1E-06	9,084	threonine-type endopeptidase activity	peripheral
GO:0001104	8E-06	3,154	RNA polymerase II transcription cofactor activity	peripheral
GO:0016772	8E-06	1,472	transferase activity, transferring phosphorus-containing groups	peripheral
GO:0003735	9E-06	2,312	structural constituent of ribosome	peripheral
GO:0005024	2E-05	12,575	transforming growth factor beta-activated receptor activity	peripheral
GO:0005024	2E-05	12,575	transforming growth factor beta-activated receptor activity	peripheral
GO:0005024	2E-05	12,575	transforming growth factor beta-activated receptor activity	peripheral
GO:0005234	2E-05	7,680	extracellular-glutamate-gated ion channel activity	peripheral

GO:0008066	3E-05	5,588	glutamate receptor activity	peripheral
GO:0008066	3E-05	5,588	glutamate receptor activity	peripheral
GO:0004715	5E-05	3,729	non-membrane spanning protein tyrosine kinase activity	peripheral
GO:0004532	0,0002	4,787	exoribonuclease activity	peripheral
GO:0030374	0,0002	3,394	ligand-dependent nuclear receptor transcription coactivator activity	peripheral
GO:0030234	0,0003	1,400	enzyme regulator activity	peripheral
GO:0003707	0,0007	2,795	steroid hormone receptor activity	peripheral
GO:0003707	0,0007	2,795	steroid hormone receptor activity	peripheral
GO:0000175	0,0007	7,821	3'-5'-exoribonuclease activity	peripheral
GO:0016796	0,0013	3,300	exonuclease activity, active with either ribo- or deoxyribonucleic acids and pro...	peripheral
GO:0034062	0,0027	2,792	RNA polymerase activity	peripheral
GO:0005085	0,0041	2,221	guanyl-nucleotide exchange factor activity	peripheral
GO:0019208	0,0045	2,149	phosphatase regulator activity	peripheral
GO:0004702	0,0046	2,096	receptor signaling protein serine/threonine kinase activity	peripheral
GO:0004702	0,0046	2,096	receptor signaling protein serine/threonine kinase activity	peripheral
GO:0022834	0,0047	1,746	ligand-gated channel activity	peripheral
GO:0004860	0,0053	2,351	protein kinase inhibitor activity	peripheral
GO:0016462	0,0059	1,283	pyrophosphatase activity	peripheral
GO:0008601	0,0065	3,718	protein phosphatase type 2A regulator activity	peripheral
GO:0004879	0,0076	2,507	ligand-activated sequence-specific DNA binding RNA polymerase II transcription f...	peripheral
GO:0016817	0,0078	1,270	hydrolase activity, acting on acid anhydrides	peripheral
GO:0004672	0,0081	1,339	protein kinase activity	peripheral
GO:0005487	0,0081	4,778	nucleocytoplasmic transporter activity	peripheral
GO:0030676	0,0081	4,778	Rac guanyl-nucleotide exchange factor activity	peripheral
GO:0015279	0,0105	5,572	store-operated calcium channel activity	peripheral
GO:0003924	0,0118	1,509	GTPase activity	peripheral
GO:0004842	0,0148	1,434	ubiquitin-protein ligase activity	peripheral
GO:0004896	0,015	1,900	cytokine receptor activity	peripheral
GO:0004896	0,015	1,900	cytokine receptor activity	peripheral
GO:0030545	0,0164	2,308	receptor regulator activity	peripheral
GO:0004535	0,0169	4,643	poly(A)-specific ribonuclease activity	peripheral
GO:0001056	0,018	3,715	RNA polymerase III activity	peripheral
GO:0004714	0,0208	1,997	transmembrane receptor protein tyrosine kinase activity	peripheral
GO:0004714	0,0208	1,997	transmembrane receptor protein tyrosine kinase activity	peripheral
GO:0004714	0,0208	1,997	transmembrane receptor protein tyrosine kinase activity	peripheral
GO:0005088	0,022	1,622	Ras guanyl-nucleotide exchange factor activity	peripheral
GO:0019887	0,0238	2,012	protein kinase regulator activity	peripheral
GO:0000989	0,0238	2,367	transcription factor binding transcription factor activity	peripheral
GO:0001054	0,0254	3,980	RNA polymerase I activity	peripheral
GO:0005230	0,0269	1,794	extracellular ligand-gated ion channel activity	peripheral
GO:0003712	0,0274	1,478	transcription cofactor activity	peripheral
GO:0030695	0,0305	1,346	GTPase regulator activity	peripheral
GO:0005099	0,0323	1,586	Ras GTPase activator activity	peripheral
GO:0016881	0,0343	1,335	acid-amino acid ligase activity	peripheral
GO:0005344	0,0363	3,482	oxygen transporter activity	peripheral

GO:0005246	0,043	2,347	calcium channel regulator activity	peripheral
GO:0003713	0,0437	1,360	transcription coactivator activity	peripheral
GO:0005057	0,044	1,923	receptor signaling protein activity	peripheral
GO:0019209	0,0456	1,912	kinase activator activity	peripheral
GO:0008138	0,0459	1,978	protein tyrosine/serine/threonine phosphatase activity	peripheral
GO:0004526	2E-06	41,197	ribonuclease P activity	ultra-peripheral
GO:0005212	0,0011	4,576	structural constituent of eye lens	ultra-peripheral
GO:0004725	0,0031	1,911	protein tyrosine phosphatase activity	ultra-peripheral
GO:0022834	0,0114	1,601	ligand-gated channel activity	ultra-peripheral
GO:0015278	0,0151	3,555	calcium-release channel activity	ultra-peripheral
GO:0004622	0,0182	3,917	lysophospholipase activity	ultra-peripheral
GO:0017017	0,0182	3,917	MAP kinase tyrosine/serine/threonine phosphatase activity	ultra-peripheral
GO:0005085	0,0213	1,453	guanyl-nucleotide exchange factor activity	ultra-peripheral
GO:0046961	0,0218	3,200	proton-transporting ATPase activity, rotational mechanism	ultra-peripheral
GO:0046961	0,0218	3,200	proton-transporting ATPase activity, rotational mechanism	ultra-peripheral
GO:0004521	0,0261	1,961	endoribonuclease activity	ultra-peripheral
GO:0016820	0,0266	1,606	hydrolase activity, acting on acid anhydrides, catalyzing transmembrane movement...	ultra-peripheral
GO:0016820	0,0266	1,606	hydrolase activity, acting on acid anhydrides, catalyzing transmembrane movement...	ultra-peripheral
GO:0010576	0,0271	3,428	metalloenzyme regulator activity	ultra-peripheral
GO:0016791	0,0278	1,359	phosphatase activity	ultra-peripheral
GO:0016289	0,0303	2,909	CoA hydrolase activity	ultra-peripheral
GO:0015267	0,0326	1,269	channel activity	ultra-peripheral
GO:0016493	0,0332	3,808	C-C chemokine receptor activity	ultra-peripheral
GO:0016493	0,0332	3,808	C-C chemokine receptor activity	ultra-peripheral
GO:0016860	0,0348	1,982	intramolecular oxidoreductase activity	ultra-peripheral
GO:0008528	0,0353	1,528	G-protein coupled peptide receptor activity	ultra-peripheral
GO:0008528	0,0353	1,528	G-protein coupled peptide receptor activity	ultra-peripheral
GO:0022892	0,038	1,166	substrate-specific transporter activity	ultra-peripheral
GO:0048018	0,0384	3,046	receptor agonist activity	ultra-peripheral
GO:0015662	0,0397	2,177	ATPase activity, coupled to transmembrane movement of ions, phosphorylative mech...	ultra-peripheral
GO:0015662	0,0397	2,177	ATPase activity, coupled to transmembrane movement of ions, phosphorylative mech...	ultra-peripheral
GO:0043492	0,0402	1,546	ATPase activity, coupled to movement of substances	ultra-peripheral
GO:0016638	0,0407	2,666	oxidoreductase activity, acting on the CH-NH2 group of donors	ultra-peripheral
GO:0090484	0,0407	2,666	drug transporter activity	ultra-peripheral
GO:0015079	0,0429	1,453	potassium ion transmembrane transporter activity	ultra-peripheral
GO:0003777	0,0449	1,634	microtubule motor activity	ultra-peripheral
GO:0016830	0,0459	1,829	carbon-carbon lyase activity	ultra-peripheral

Supplementary Table S5: Distribution of disease-associated germline and cancer somatic mutations in a set of driver genes also associated to Mendelian diseases.

Gene	symbol	UniProt	cds	NSD	NSnD	NnSD	NnSnD	OR	p-value	set	inheritance
27034	ACAD8	Q9UKU7	415	2	15	10	388	0,133	2,2E-03	OG	NA
60	ACTB	P60709	375	1	31	14	329	0,032	1,6E-08	OG	AD
71	ACTG1	P63261	375	0	39	15	321	0,000	8,2E-12	OG	AD
324	APC	P25054	2843	17	267	21	2538	0,064	1,7E-56	TS	AD
472	ATM	Q13315	3056	10	195	66	2785	0,051	1,7E-44	TS	AR
546	ATRX	P46100	2492	7	200	25	2260	0,035	1,2E-48	TS	NA
641	BLM	P54132	1417	0	80	12	1325	0,000	4,7E-24	TS	AR
659	BMPR2	Q13873	1038	1	47	19	971	0,021	3,3E-13	TS	AD
673	BRAF	P15056	766	8	45	22	691	0,178	3,4E-07	OG	AD
675	BRCA2	P51587	3418	4	161	19	3234	0,025	6,4E-42	TS	AD
124583	CANT1	Q8WVQ1	401	1	19	9	372	0,053	3,6E-05	OG	AR
11200	CHEK2	O96017	543	0	27	16	500	0,000	1,3E-08	TS	AD
1123	CHN1	P15882	459	0	30	8	421	0,000	1,8E-09	OG	NA
1277	COL1A1	P02452	1464	8	87	123	1246	0,092	2,0E-17	OG	AD
1387	CREBBP	Q92793	2442	9	163	28	2242	0,055	7,6E-37	TS	AD
1499	CTNNB1	P35222	781	5	45	2	729	0,111	7,1E-09	OG	AD
23405	DICER1	Q9UPY3	1922	1	86	3	1832	0,012	2,9E-24	TS	AD
1785	DNM2	P50570	870	3	37	14	816	0,081	1,7E-08	TS	AD
2146	EZH2	Q15910	746	2	50	4	690	0,040	1,2E-12	TS	AD
355	FAS	P25445	335	1	19	17	298	0,053	3,5E-05	TS	AD
2260	FGFR1	P11362	822	3	33	67	719	0,091	2,0E-07	OG	AD
2263	FGFR2	P21802	821	4	62	37	718	0,065	3,7E-14	OG	AD
2261	FGFR3	P22607	806	3	34	18	751	0,088	1,1E-07	OG	AD
2335	FN1	P02751	2386	0	136	5	2245	0,000	9,0E-41	TS	AD
668	FOXL2	P58012	376	0	7	23	346	0,000	1,5E-02	OG	AD
2623	GATA1	P15976	413	0	21	8	384	0,000	8,4E-07	OG	NA
2624	GATA2	P23769	480	0	19	6	455	0,000	3,4E-06	OG	AD
2625	GATA3	P23771	443	0	77	6	360	0,000	4,3E-22	TS	AD
2767	GNA11	P29992	359	1	11	4	343	0,091	6,0E-03	OG	NA
2778	GNAS	Q5JWF2	1037	4	61	7	965	0,066	6,6E-14	OG	AD
3239	HOXD13	P35453	343	0	17	5	321	0,000	1,4E-05	OG	AD
3265	HRAS	P01112	189	2	7	5	175	0,286	1,7E-01	OG	AD
3636	INPPL1	O15357	1258	1	66	4	1187	0,015	1,1E-18	TS	NA
3762	KCNJ5	P48544	419	0	33	5	381	0,000	2,5E-10	OG	NA
8242	KDM5C	P41229	1560	0	78	14	1468	0,000	9,3E-24	TS	NA
3815	KIT	P10721	976	2	61	9	904	0,033	8,4E-16	OG	AD
8085	KMT2D	O14686	5537	9	331	31	5166	0,027	9,5E-84	TS	AD
3845	KRAS	P01116	189	6	19	7	157	0,316	1,3E-02	OG	AD
3908	LAMA2	P24043	3122	5	242	10	2865	0,021	4,4E-63	TS	AR
9935	MAFB	Q9Y5Q3	323	0	14	8	301	0,000	1,1E-04	OG	AD
9968	MED12	Q93074	2177	1	108	4	2064	0,009	8,2E-31	OG	NA
4221	MEN1	O00255	615	1	27	8	579	0,037	1,9E-07	TS	AD
4286	MITF	O75030	526	0	25	9	492	0,000	5,3E-08	OG	AD
3110	MNX1	P50219	401	0	7	11	383	0,000	1,5E-02	OG	AD
4352	MPL	P40238	635	0	22	8	605	0,000	4,2E-07	OG	AR
4613	MYCN	P04198	464	0	28	5	431	0,000	6,8E-09	OG	AD
4629	MYH11	P35749	1972	1	109	6	1856	0,009	4,4E-31	OG	AD
79784	MYH14	Q7Z406	1995	0	76	10	1909	0,000	2,9E-23	OG	AD

4627	MYH9	P35579	1960	0	94	14	1852	0,000	3,1E-28	OG	AD
4763	NF1	P21359	2839	20	256	70	2493	0,078	2,7E-51	TS	AD
4771	NF2	P35240	595	4	34	20	537	0,118	5,5E-07	TS	AD
4893	NRAS	P01111	189	6	10	0	173	0,600	4,4E-01	OG	AD
4914	NTRK1	P04629	796	6	57	12	721	0,105	2,5E-11	OG	AD
5077	PAX3	P23760	479	2	33	20	424	0,061	3,6E-08	OG	AD
7849	PAX8	Q06710	450	0	25	7	418	0,000	5,3E-08	OG	AR
5159	PDGFRB	P09619	1106	1	68	3	1034	0,015	5,2E-19	OG	AD
84295	PHF6	Q8IWS0	365	3	34	6	322	0,088	2,0E-07	TS	NA
5290	PIK3CA	P42336	1068	15	78	7	968	0,192	3,9E-11	OG	AD
5468	PPARG	P37231	505	3	22	7	473	0,136	1,4E-04	OG	AD
5573	PRKAR1A	P10644	381	0	24	14	343	0,000	1,1E-07	TS	AD
10594	PRPF8	Q6P2Q9	2335	0	85	7	2243	0,000	6,0E-26	OG	AD
5727	PTCH1	Q13635	1447	0	93	17	1337	0,000	5,7E-28	TS	AD
5728	PTEN	P60484	403	36	169	10	188	0,213	3,6E-15	TS	AD
5781	PTPN11	Q06124	597	12	27	27	531	0,444	2,2E-02	OG	AD
5894	RAF1	P04049	648	1	18	19	610	0,056	7,0E-05	OG	NA
5979	RET	P07949	1114	7	57	57	993	0,123	1,2E-10	OG	AD
6103	RPGR	Q92834	1020	1	46	21	952	0,022	6,5E-13	TS	NA
10801	SEPT9	Q9UHD8	586	0	14	4	568	0,000	1,1E-04	OG	AD
26040	SETBP1	Q9Y6X0	1596	4	120	5	1467	0,033	4,5E-30	OG	AD
4089	SMAD4	Q13485	552	5	88	2	457	0,057	2,2E-19	TS	AD
8243	SMC1A	Q14683	1233	6	51	18	1158	0,118	5,2E-10	OG	NA
6654	SOS1	Q07889	1333	8	57	28	1240	0,140	5,1E-10	OG	AD
6662	SOX9	P48436	509	0	40	12	457	0,000	2,2E-12	TS	AD
6774	STAT3	P40763	770	3	40	16	711	0,075	2,8E-09	OG	AD
6794	STK11	Q15831	433	3	47	11	372	0,064	1,3E-10	TS	AD
6926	TBX3	O15119	743	1	63	3	676	0,016	1,6E-17	TS	AD
7048	TGFBR2	P37173	567	2	58	22	485	0,034	1,3E-14	TS	AD
7157	TP53	P04637	393	63	196	4	130	0,321	1,6E-09	TS	AD
7248	TSC1	Q92574	1164	0	44	13	1107	0,000	1,0E-13	TS	AD
7249	TSC2	P49815	1807	3	75	51	1678	0,040	1,1E-18	TS	AD
7428	VHL	P40337	213	48	51	22	92	0,941	9,0E-01	TS	AD

Gene: Entrez gene identifier, **Symbol**: official gene symbol **cds**: length of the coding sequence, **UniProt**: UniProt Accession, **nSD**: number of positions bearing cancer somatic mutations and disease germline mutations, **nSnD**: number of positions bearing only cancer somatic mutations, **nnSD**: number of positions bearing only disease germline mutations, **nnSnD**: number of positions without known mutations, **OR**: Fisher test odds ratio, **p-value**: p-value of the Fisher test, driver type: tumor suppressor (TS) or oncogene (OG), **inheritance**: autosomal dominant (AD), autosomal recessive (AR) or not assigned (NA)
